# Foliar spray of silica improved water stress tolerance in rice (*Oryza sativa* L.) cultivars

**DOI:** 10.3389/fpls.2022.935090

**Published:** 2022-11-16

**Authors:** Samira A. F. El-Okkiah, Mohamed M. El-Afry, Safaa A. Shehab Eldeen, Amira M. El-Tahan, Omar M. Ibrahim, Mostafa M. Negm, Mohamad Alnafissa, Mohamed T. El-Saadony, Hessa M. R. S. Almazrouei, Synan F. AbuQamar, Khaled A. El-Tarabily, Dalia A. Selim

**Affiliations:** ^1^ Agricultural Botany Department, Faculty of Agriculture, Kafrelsheikh University, Kafr El-Sheikh, Egypt; ^2^ Plant Production Department, Arid Lands Cultivation Research Institute, The City of Scientific Research and Technological Applications (SRTA-City), Borg El Arab, Alexandria, Egypt; ^3^ Department of Agricultural Economics, Faculty of Agriculture, Al-Azhar University, Cairo, Egypt; ^4^ Department of Agricultural Economics, College of Food and Agriculture Sciences, King Saud University, Riyadh, Saudi Arabia; ^5^ Department of Agricultural Microbiology, Faculty of Agriculture, Zagazig University, Zagazig, Egypt; ^6^ Department of Biology, College of Science, United Arab Emirates University, Al Ain, United Arab Emirates; ^7^ Khalifa Center for Genetic Engineering and Biotechnology, United Arab Emirates University, Al Ain, United Arab Emirates; ^8^ Harry Butler Institute, Murdoch University, Murdoch, WA, Australia; ^9^ Department of Agricultural Botany, Faculty of Agriculture, Menoufia University, Shibin El-Kom, Egypt

**Keywords:** Drought tolerance, *Oryza sativa*, root anatomy, proline, peroxidase, silica

## Abstract

Rice (*Oryza sativa* L.) is a major cereal crop and a staple food across the world, mainly in developing countries. Drought is one of the most important limiting factors for rice production, which negatively affects food security worldwide. Silica enhances antioxidant activity and reduces oxidative damage in plants. The current study evaluated the efficiency of foliar spray of silica in alleviating water stress of three rice cultivars (Giza178, Sakha102, and Sakha107). The seedlings of the three cultivars were foliar sprayed with 200 or 400 mg l^-1^ silica under well-watered [80% water holding capacity (WHC)] and drought-stressed (40% WHC)] conditions for two summer seasons of 2019 and 2020. The obtained results demonstrated that drought stress caused significant decreases in growth, yield, and physiological parameters but increases in biochemical parameters (except proline) of leaves in all rice cultivars compared to well-irrigated plants (control). The roots of drought-stressed seedlings exhibited smaller diameters, fewer numbers, and narrower areas of xylem vessels compared to those well-watered. Regardless of its concentration, the application of silica was found to increase the contents of photosynthetic pigments and proline. Water relation also increased in seedlings of the three tested rice cultivars that were treated with silica in comparison to their corresponding control cultivars when no silica was sprayed. Foliar application of 400 mg l^-1^ silica improved the physiological and biochemical parameters and plant growth. Overall, foliar application of silica proved to be beneficial for mitigating drought stress in the tested rice cultivars, among which Giza178 was the most drought-tolerant cultivar. The integration of silica in breeding programs is recommended to improve the quality of yield and to provide drought-tolerant rice cultivars under drought-stress conditions.

## 1 Introduction

Rice (*Oryza sativa* L.) is the second most critical commercially farmed cereal crop in the world. More than half of the world’s people rely on rice for their primary nutrition and energy (Muthayya et al., 2014). Thus, more rice is needed to achieve food security and satisfy the increasing demand of the growing population. Growing rice in flood irrigation systems accounts for over 75% of the global production (FAOSTAT, 2017). In contrast with other crops, rice is relatively more sensitive to water scarcity (drought) especially at critical growth stages (Panda et al., 2021). Drought stress, at the flowering stage, has a strong influence on rice physiological traits and yield (Panda et al., 2021).

In general, drought is one of the most devastating climate events that threaten agricultural production worldwide. Water deprivation inhibits cell division, resulting in short stems, reduced internodal length, truncated tilling capability, and a compromised root system (Hannan et al., 2020) and causes reduction in dry and fresh biomass (Sikuku et al., 2012). It has been reported that drought stress causes varying root lengths, altered root morphology, and reduced root development in rice plants (Kim et al., 2020). In addition, many metabolic processes, including photosynthesis, respiration, ion absorption, development of hormones, and nutrient uptake are negatively affected by drought stress conditions (Farooq et al., 2008; Usman et al., 2013; Lee et al., 2015). Drought stress may also cause considerable damages to photosynthetic pigments, gas exchange systems, electron transport systems, photosystems, carbon reduction routes, and enzyme systems (Ashraf and Harris, 2013). In rice, water deficiency typically occurs in leaves resulting in the loss of chlorophylls (Chl) *a* and *b* and carotenoids that are essential for photosynthesis (Farooq et al., 2009). Generally, drought stress is one such abiotic stress which causes major setbacks to agricultural productivity. Thus, cereal crops (e.g., rice) have contrasting adaptive responses to cope with drought (Javaid et al., 2022).

Plants display a variety of morphological, physiological, biochemical, and molecular attributes to mitigate the effects of drought stress. Such morphological mechanisms of drought avoidance and phenotypic flexibility can help crop plants to survive under drought stress (Choudhary et al., 2009). The architecture of the root system allows reserve of more water quantity for drought tolerance (Choudhary et al., 2009). Cell and tissue water preservation, cell membrane stability, and endogenously produced growth regulators are some of the physiological mechanisms associated with plant response to drought stress conditions. At the molecular level, plants alter gene expression to avoid hazardous effects of low water availability. Thus, these adaptive responses are controlled by genetic factors at different stages of plant growth (Choudhary et al., 2009). Relative water content (RWC) is an important indicator of water status and represents a screening tool for drought tolerance in plants (Liang et al., 2007; Choudhary et al., 2009). In rice, the leaf rolling factor under drought stress is considered as one of the best criteria to estimate the levels of drought tolerance in a large-scale screening (Pandey and Shukla, 2015).

Silica has been widely used in improving plant tolerance against environmental stresses. Although it is not classified as an essential element for plants, it has beneficial effects in alleviating diverse forms of abiotic and biotic stresses (Liang et al., 2007; Hamayun et al., 2010). Many studies have reported that application of silica on plants can not only activate the plant defense system but also regulate RWC, net photosynthetic ratio, intercellular CO_2_ level, stomatal conductance, and transpiration ratio (Romero-Aranda et al., 2006; Gong et al., 2008; Chen et al., 2016; Hussain et al., 2021). In addition, silica plays a vital role in improving the physiological activities and enhancing the cellular metabolic rates in plants in response to drought stress, thus enhancing water use efficiency, growth, and biomass (Gong et al., 2003; Ahmad et al., 2007; Li et al., 2018). 

In the present study, we hypothesized that the rice cultivar Giza178 in Egypt could be a potential drought-tolerant cultivar combined with high yielding upon the application of silica under drought stress conditions. Therefore, the current study aimed to evaluate the level of drought tolerance among the three Egyptian rice cultivars (Giza178, Sakha102, and Sakha107) sprayed with two dosages of silica (200 or 400 mg l^-1^) *via* assessing the morphophysiological and biochemical parameters, including productivity, photosynthetic pigments, RWC, proline content, and total antioxidant activity. This study could also provide basic principles useful for the management of phenotyping practices for the genetic dissection of drought tolerance and hence the release of drought-tolerant rice cultivars.

## 2 Materials and methods

### 2.1 Soil analyses of the experimental site

Soil samples were taken from the major root zone before rice was planted at the end of the two growing seasons. The soil samples were air-dried, crushed, passed through a 2-mm sieve, and analyzed for various physicochemical properties. Soil texture was determined using the hydrometer method (Jackson, 1973). Rice was the preceding crop in both seasons. The type and chemical and physical characteristics of the soil are presented in **Table (S1)**.

### 2.2 Plant material and treatments

Silica, in the form of potassium silicate 10% K_2_O and 25% SiO_2_ (Sigma-Aldrich Chemie GmbH, Taufkirchen, Germany) sprayed on leaves of three native rice cultivars (Giza178, Sakha102, and Sakha107), were evaluated in response to drought stress. Rice plants were foliar sprayed twice during the vegetative growth stage [100 and 120 days after sowing (DAS)] with the aqueous potassium silicate solutions at 200 and 400 mg l^-1^. These cultivars were provided by the Agricultural Research Center, Rice Research and Training Center, Kafr El-Sheikh, Egypt. This study was carried out at Sakha Agricultural Research Station, Kafr El-Sheikh, Egypt, using the lysimeter technique during two summer rice growing seasons 2019 and 2020. The Lysimeter consisted of concrete beds (1 meter width x 2 meter length x 1 meter depth) filled with soil to a depth of 100 cm in three layers: 60 cm clay at the surface, 20 cm sand in the middle, and 20 cm gravel at the bottom. Seeds were surface sterilized with 2% sodium hypochlorite for 5 min, followed by multiple rinses with sterile water. Seeds of the rice cultivars were separately sown in plots containing equivalent quantities of homogenous clay:sand (2:1) on May 15 and 19 of the first and second seasons, respectively.

All plants in plots received the recommended dose of NPK fertilizers. Nitrogen (N) was applied in three split doses: the first split dose of 30 kg ha^-1^ N was applied as basal application along with the full dose of phosphorus (30 kg ha^-1^) and potassium (30 kg ha^-1^), followed by top dressing of two split doses of 15 kg ha^-1^ N each at the tillering and panicle initiation stages. Seeds were sown manually by the dibbling method maintaining plant spacing of 20 × 15 cm. A split–split plot model was applied in this study with three replications. The pedigree, salience, and feature of rice genotypes used in the study are listed in **Table (S2)**.

The performance of the three rice cultivars was evaluated under normal (well-irrigated) conditions [80% water-holding capacity (WHC)] or drought stress (40% WHC). All plots received adequate irrigation until 30 days before transplantation. Then, by withholding water from half of each group, drought stress was imposed (irrigation every 10 days) for 5 months. Drought tolerance was defined as the ability of plants to survive under drought conditions, endure drought without injury, or be efficient in their use of water (Arnon, 1972), and upon the foliar application of silica at the concentration of 200 or 400 mg l^-1^. Plants with no silica treatment served as control.

### 2.3 Morphological characteristics and yield parameters

At 60 DAS, growth parameters, such as plant height (PH, cm), root volume (RV, cm^3^), and flag leaf area (FLA) plant^-1^ (cm^2^), were measured using a portable meter.

At maturity (150 DAS), the plants from each treatment were individually harvested, and their grains were manually counted to measure the 1000 grain weight or grain index (GI, g) of each treatment. Yield components, including the number of tillers, panicle length (PL; cm), and panicle weight (PW; g), were determined. From each treatment, three plants were chosen and separated by their tillers to manually estimate their average values.

### 2.4 Physiological and biochemical parameters

#### 2.4.1 Assessment of photosynthetic pigments

In both seasons, the photosynthetic pigments, Chl *a* and *b* and total Chl, were determined using the fourth leaf from the tip of the rice plants at 60 DAS. Chl concentrations were calculated as μg cm^-2^ fresh area of 1 cm^2^ of the leaf. The pigments were extracted with 5 ml of N,N-dimethylformamide before being stored in the refrigerator for 24 h in the dark. Absorbance at wavelengths of 664 and 647 nm in samples were estimated by the spectrophotometer (UV-2101/3101 PC; Shimadzu Corporation, Analytical Instruments Division, Kyoto, Japan). The photosynthetic pigment level was determined according to Moran (1982), with the following equations and the pigment contents were calculated as µg cm^-2^:


Chl a (µg ml−1) = 12.64 A664 − 2.99 A647

Chl b (µg ml−1) = 23.26 A664 − 5.6 A647

Total Chl (µg ml−1) = 7.04 A664 + 20.27 A647

#### 2.4.2 Assessment of RWC

The fresh weight (FW) of the second youngest leaf was used to determine the RWC in leaves. This was done by removing 1-cm disks from the upper part leaf of each plant, where remotely sensed data were collected. The five disks were immediately weighed, providing a measure of FW. The disks were then soaked in deionized water for 24 h before being weighed again to obtain a fully turgid weight (TW). Finally, the leaf disks were dried at 85°C and weighed to determine their dry weight (DW). The RWC of a leaf was calculated according to the equation provided by Salisbury and Ross (1992):


RWC = (FW − DW) / (TW − DW) × 100

#### 2.4.3 Anatomical features of rice tissues

A minimum of five samples of roots and stems were taken at random 10 days after the application of silica. One cm-long specimens were taken from the fourth upper internode. The sampled material was fixed for 48 h, in formaldehyde:alcohol: acetic acid (FAA) solution (50% ethanol + 5% formaldehyde + 10% glacial acetic acid in water). Two washes in 70% ethyl alcohol were performed on the samples. Dehydration was achieved by passing the samples through a series of ethyl alcohol concentrations (75%–100%). Each sample was passed through a mixture of xylol and absolute ethyl alcohol in the following percentages: 25%, 50%, and 75%, and pure xylol in the final two changes for each dilution. Within 12 h, a paraffin shaving reagent containing samples was saturated. To remove all traces of xylol, two changes of paraffin were performed. Samples were immersed in melted paraffin in embedding paper trays, and then quickly cooled in cold water.

Rotary Microtome (Leica RM 2125, Vienna, Austria) sections (10–12 µm thick) were cut, and paraffin sections were fixed to the slides with albumin. Slides were dried completely in a dry oven at 50°C for 24 h. The slides were first immersed in two changes of xylol for about 10 s before being transferred to a jar containing equal parts of absolute ethyl alcohol and xylol for 5 min. The sections were immersed in a series of descending ethyl alcohol dilutions ranging from absolute to 5%. The sections were stained for 10 min in a jar containing 1% safranin, and the excess stain was washed away. Sections were then stained in a jar containing 1% light green for 1 min, then cleared in xylol, mounted in Canada balsam (Ruzin, 1999). Samples were examined using a Olympus BH-2 (Olympus Optical Co., Ltd., Tokyo, Japan) light microscope equipped with a digital camera and software (Jenoptik ProgRes Camera, C12plus, Frankfurt, Germany).

#### 2.4.4 Measurement of proline

The amount of free proline was estimated as described by Bates et al. (1973). Half-gram FW of plant cells was homogenized in 10 ml of 3% sulfosalicylic acid (Sigma-Aldrich), and the homogenate was filtered using Whatman No. 1 filter paper (Whatman, Maidstone, England). The filtrate was made up to 10 ml, and 2 ml of the filtrate was mixed with 2 ml of ninhydrin reagent (Sigma-Aldrich) and glacial acetic acid (Sigma-Aldrich).

After 1 h of incubation, the mixture was immersed in a boiling water bath. Subsequently, the mixture was cooled in an ice bath. The reaction mixture was then extracted with 4 ml of toluene (Sigma-Aldrich) and vigorously shaken for 15–20 s. The mixture was separated in a separating funnel. The upper phase was taken, and absorbance was determined at 520 nm. Toluene was used as a control. The proline level was calculated as g g^-1^ FW *via* a proline standard calibration curve.

#### 2.4.5 Measurement of malondialdehyde content

The lipid peroxidation level was determined using the malondialdehyde (MDA) measurement method (Heath and Packer, 1968). Leaf samples (1 g FW) were homogenized in 10 ml of trichloroacetic acid (Sigma-Aldrich). The homogenate was centrifuged for 5 min at 15,000 × g. A 1-ml aliquot of the supernatant was mixed with 4 ml of thiobarbituric acid containing 20% (w/v) trichloroacetic acid (Sigma-Aldrich). The mixture was heated at 95°C for 30 min before cooling quickly in an ice bath and then centrifuged at 10,000 × *g* for 10 min.

Using the spectrophotometer (Shimadzu Corporation), the absorbance of the supernatant was recorded at 532 and 600 nm. The MDA level was calculated by multiplying the absorbance difference (A532–A600) by the molar extinction coefficient (155 mM^−1^ cm^−1^) and the results were expressed in nmol g^-1^ FW.

#### 2.4.6 Assays of the enzymatic antioxidant compounds

The catalase (CAT; EC: 1.11.1.6) activity (U mg^–1^ protein) was assayed according to the method described by Aebi et al. (1974), whereas peroxidase (POD; EC: 1.11.1.17) activity (U mg^–1^ protein) was measured as described by Pütter (1974). The total soluble protein content of the enzyme extracts from leaves was determined following the method of Bradford (1976), using bovine serum albumin (BSA) as a protein standard.

#### 2.4.7 Assays of the total antioxidant compounds

Seed powder (100 mg) was mixed with 2 ml of methanol (Sigma-Aldrich), and the mixture was kept overnight at room temperature to determine the radical scavenging activity. One milliliter of the filtrate was added to 3 ml of 0.1 mM of 2,2-diphenyl-1-picrylhydrazyl (DPPH) (Sigma-Aldrich) and incubated for 30 min in the dark. Absorbance at 515 nm was measured with a spectrophotometer (Shimadzu Corporation), and the percentage of DPPH scavenging activity was calculated as previously mentioned by Sullivan and Ross (1979) using the subsequent formula:


$$DPPH\;activity\;\left(\%\right)\;=\;\left(Absorbance_{control}\;-\;absorbance_{sample}\right)\;/\;Absorbance_{control}\times \;100$$

### 2.5 Statistical analysis

All data were expressed as the means with the standard error of three replicates and analyzed by analysis of variance (ANOVA) using SPSS software (version 13.0; SPSS Inc., Chicago, USA). Statistical differences between treatments within the same rice line were determined using the least significant difference (LSD) at a 0.05 probability level. Correlation analysis was carried out using the Spearman coefficient between each pair of the studied traits over control and drought stress separately, as well as all over the two treatments in the upper triangle of the produced plot. Path analysis was determined using the R statistical software version 4.1.0, (R Core Team, 2021) using the (lavaan) package and the function (sem), which stands for structural equation modeling (Tabachnick and Fidell, 1996). A path diagram was drawn by using the (semPaths) function in the same package. The results of the path analysis are shown on the path diagrams. Heatmap was produced to show the relation among the treatments and the studied traits on the base of color scale using the standardized data by subtracting the mean from each value and divided by the standard deviation.

## 3 Results

### 3.1 Effects of drought stress and silica application on growth and yield of rice

The morphological features of the three rice cultivars, in terms of PH, FLA, and RV, were affected by drought (**Tables 1**, **S3**). At vegetative and flowering stages, drought considerably lowered the PH in all the studied cultivars. Under drought stress, the cultivars Sakha107 and Sakha102 had the least noticeable decrease in PH with 27.8% and 27.9%, respectively, less than its corresponding cultivar under well-watered (control) conditions, followed by Giza178 with a 11.17% and 11.9 decrease more diminutive than the control, in both seasons (**Table 1**).

**Table 1:** Effects of foliar application of silica on some morphological parameters at vegetative stage in response to drought stress during the growing seasons of 2019 and 2020.

Treatment		Cultivar	PH (cm)		FLA (cm^2^)		RV (cm^3^)	
Water status	Silica (mg l^-1^)		2019	2020	2019	2020	2019	2020
**Well-irrigated (80% WHC)**	**0**	**G178**	95.17 ± 0.6^def^	93.17 ± 0.6^e^	18.24 ± 0.57^de^	18.49 ± 0.57^de^	60.14 ± 0.58^f^	60.81 ± 0.58^f^
		**SK102**	102.00 ± 0.58^ab^	102.17 ± 0.6^a^	17.60 ± 0.06^ef^	18.10 ± 0.06^de^	28.33 ± 0.50^k^	29.83 ± 0.51^k^
		**SK107**	98.58 ± 0.46^bcd^	97.67 ± 0.42^cd^	17.92 ± 0.27^de^	18.30 ± 0.28^de^	44.24 ± 0.05^j^	45.32 ± 0.05^j^
	**200**	**G178**	97.00 ± 0.58^cde^	95.00 ± 0.58^de^	20.21 ± 0.59^c^	20.46 ± 0.59^c^	67.33 ± 0.60^d^	68.00 ± 0.60^d^
		**SK102**	101.67 ± 0.88^ab^	101.67 ± 0.88^ab^	18.48 ± 0.10^de^	18.98 ± 0.10^d^	51.00 ± 0.58^h^	52.50 ± 0.58^h^
		**SK107**	99.33 ± 0.17^bc^	98.33 ± 0.17^bcd^	19.35 ± 0.34^cd^	19.72 ± 0.34^d^	59.17 ± 0.58^f^	60.25 ± 0.58^f^
	**400**	**G178**	103.00 ± 0.58^a^	101.00 ± 0.58^abc^	30.06 ± 0.06^a^	30.31 ± 0.06^a^	71.50 ± 0.58^c^	72.17 ± 0.58^c^
		**SK102**	104.00 ± 0.58^a^	104.00 ± 0.58^a^	18.90 ± 0.02^cde^	19.40 ± 0.02^d^	47.66 ± 0.06^i^	49.16 ± 0.06^i^
		**SK107**	103.50 ± 0.50^a^	102.50 ± 0.50^a^	24.48 ± 0.04^b^	24.86 ± 0.04^b^	59.58 ± 0.28^f^	60.67 ± 0.28^f^
**Drought (40% WHC)**	**0**	**G178**	84.00 ± 0.58^h^	82.00 ± 0.58^g^	18.27 ± 0.64^de^	18.52 ± 0.64^de^	71.00 ± 0.58^c^	71.67 ± 0.58^c^
		**SK102**	73.67 ± 0.88^j^	73.67 ± 0.88^l^	12.43 ± 0.23^i^	12.93 ± 0.23^h^	22.29 ± 0.00^l^	23.79 ± 0.10^l^
		**SK107**	78.83 ± 0.73^i^	77.83 ± 0.73^h^	14.82 ± 0.45^gh^	15.73 ± 0.38^fg^	46.65 ± 0.26^i^	47.73 ± 0.26^j^
	**200**	**G178**	84.17 ± 0.73^h^	82.17 ± 0.73^j^	18.28 ± 0.36^de^	18.53 ± 0.36^de^	76.33 ± 0.88^b^	77.00 ± 0.88^b^
		**SK102**	88.50 ± 0.29^g^	88.50 ± 0.29^f^	14.11 ± 0.00^h^	14.61 ± 0.00^g^	47.29 ± 0.10^i^	48.79 ± 0.10^i^
		**SK107**	86.33 ± 0.33^gh^	85.33 ± 0.33^fg^	16.20 ± 0.10^fg^	16.57 ± 0.18^f^	61.81 ± 0.48^e^	62.90 ± 0.49^e^
	**400**	**G178**	97.00 ± 0.58^cde^	95.00 ± 0.58^de^	20.19 ± 0.71^c^	20.44 ± 0.71^c^	87.67 ± 0.64^a^	88.34 ± 0.64^a^
		**SK102**	92.5 ± 1.89^f^	92.50 ± 1.89^e^	14.73 ± 0.10^gh^	15.23 ± 0.10^fg^	55.07 ± 0.01^g^	56.51 ± 0.01^g^
		**SK107**	94.75 ± 1.09^ef^	93.75 ± 1.09^e^	17.47 ± 0.40^ef^	17.84 ± 0.40^d^	71.34 ± 0.32^c^	72.43 ± 0.32^c^

Data are presented as mean ± SE. Within columns, values followed by the same letter are not significantly (P>0.05) different. WHC, water-holding capacity; G178, Giza178; SK102, Sakha102; SK107, Sakha107; PH, plant height; FLA, flag leaf area; RV, root volume.

Furthermore, drought stress had an adverse impact on FLA of Sakha102, with 3.11%, and 3.07% decrease in FLA, respectively, compared with the control in both seasons (**Table 1**). For the RV trait, cultivars Sakha102, Giza178, and Sakha107 were arranged in ascending order (12.62%, 11.12%, and 7.65%, respectively). Moreover, drought treatment has a negative impact on RV (**Tables 1**, **S3**). In general, Giza178 and Sakha107 were the least affected by drought, followed by Sakha102 in both seasons.

Exposure to drought stress disturbs all these factors in plants; however, application of silica may mitigate the negative effect. Spraying with 400 mg l^-1^ silica resulted in better plant development factors (PH, FLA, and PW) than the other silica treatments. Regarding PH in both seasons, there was a significant interaction between rates of silica application and rice genotypes (**Table 1**). It was evident that the Sakha102 cultivar with no silica treatment produced the shortest plants. However, the cultivar Giza178 was the tallest when 400 mg l^-1^ silica was applied in both growing seasons. In response to drought stress, Giza178 cultivar sprayed with 400 mg l^-1^ silica showed the best performance in PH (124.33 and 123.00 cm in 2019 and 2020, respectively) and FLA (21.87 and 22.24 cm^2^ in 2019 and 2020, respectively) at the flowering stage, (**Tables 1**, **S3**), and PW (136.60 g in 2019 and 133.37 g in 2020) (**Table 2**).

**Table 2:** Effects of foliar application of silica on some yield components in response to drought stress during the growing seasons of 2019 and 2020.

Treatment		Cultivar	PW (g)		PL (cm)		Number of productive tillers	
Water status	Silica (mg l^-1^)		2019	2020	2019	2020	2019	2020
**Well-watered (80% WHC)**	**0**	**G178**	125.17 ± 0.64^b^	124.30 ± 0.65^b^	22.60 ± 0.61^b^	23.50 ± 0.52^b^	15.67 ± 0.33^ij^	18.67 ± 0.33^gh^
		**SK102**	88.07 ± 0.01^j^	89.51 ± 0.01^j^	19.50 ± 0.06^ef^	21.00 ± 0.06^deg^	13.67 ± 0.33^kl^	14.67 ± 0.33^j^
		**SK107**	106.59 ± 0.32^g^	106.91 ± 0.33^g^	21.05 ± 0.28^cd^	22.25 ± 0.23^b-e^	14.67 ± 0.17^jk^	16.33 ± 0.33^i^
	**200**	**G178**	117.73 ± 0.64^e^	116.73 ± 0.64^d^	21.60 ± 0.00^bcd^	22.40 ± 0.00^bcd^	26.33 ± 0.33^b^	28.33 ± 0.33^b^
		**SK102**	106.95 ± 0.75^g^	108.45 ± 0.75^fg^	19.00 ± 0.58^efg^	20.50 ± 0.58^fgh^	22.67 ± 0.33^d^	23.67 ± 0.33^e^
		**SK107**	112.34 ± 0.67^f^	112.59 ± 0.67^e^	20.30 ± 0.29^cde^	21.45 ± 0.29^c-g^	24.50 ± 0.29^c^	25.67 ± 0.33^c^
	**400**	**G178**	118.73 ± 0.64^e^	117.73 ± 0.64^d^	24.28 ± 0.36^a^	25.08 ± 0.36^a^	21.33 ± 0.33^e^	24.33 ± 0.33^de^
		**SK102**	108.38 ± 0.05^g^	109.88 ± 0.05^f^	17.77 ± 0.06^gh^	19.27 ± 0.06^hi^	18.00 ± 0.00^g^	19.00 ± 0.00^gh^
		**SK107**	113.56 ± 0.32^f^	113.81 ± 0.32^e^	21.02 ± 0.21^cd^	22.18 ± 0.21^b-e^	19.67 ± 0.17^f^	21.33 ± 0.33f
**Drought (40% WHC)**	**0**	**G178**	121.47 ± 0.64^cd^	120.47 ± 0.64^c^	19.50 ± 0.00^ef^	20.30 ± 0.00^gh^	16.67 ± 0.33^hi^	19.67 ± 0.33^g^
		**SK102**	72.74 ± 0.07^l^	74.24 ± 0.07^l^	16.74 ± 0.01^hi^	18.24 ± 0.01^ij^	8.67 ± 0.33^m^	9.67 ± 0.33^k^
		**SK107**	97.10 ± 0.36^i^	97.35 ± 0.36^l^	18.12 ± 0.00^fgh^	19.27 ± 0.00^hi^	12.67 ± 0.33^l^	14.67 ± 0.33^j^
	**200**	**G178**	122.33 ± 0.64^c^	121.33 ± 0.64^c^	21.74 ± 0.61^bc^	22.54 ± 0.61^bc^	20.33 ± 0.33^ef^	23.33 ± 0.33^e^
		**SK102**	75.91 ± 0.64^k^	77.41 ± 0.64^k^	16.22 ± 0.06^i^	17.72 ± 0.06^j^	13.00 ± 0.00^l^	14.00 ± 0.00^j^
		**SK107**	99.12 ± 0.56^i^	99.37 ± 0.56^i^	18.98 ± 0.32^efg^	20.13 ± 0.32^gh^	16.67 ± 0.17^hi^	18.33 ± 0.33^gh^
	**400**	**G178**	136.6 ± 0.64^a^	135.60 ± 0.64^a^	21.10 ± 0.49^cd^	21.90 ± 0.49^c-f^	29.00 ± 0.00^a^	32.00 ± 0.00^a^
		**SK102**	102.94 ± 0.65^h^	104.44 ± 0.65^h^	19.27 ± 0.64^efg^	20.77 ± 0.64^efg^	17.00 ± 0.00^gh^	18.00 ± 0.00^h^
		**SK107**	119.77 ± 0.01^de^	120.02 ± 0.01^c^	20.19 ± 0.32^de^	21.34 ± 0.32^c-g^	23.00± 0.00^d^	25.00 ± 0.00^cd^

Data are presented as mean ± SE. Within columns, values followed by the same letter are not significantly (P > 0.05) different. WHC, water-holding capacity; G178, Giza178; SK102, Sakha102; SK107, Sakha107; PW, panicle weight; PL, panicle length.

Drought significantly reduced yield and yield elements in any of the three rice cultivars (**Table 2**). Drought stress had negative effects on yield-contributing traits, such as PW, PL, and the number of productive tillers. In both seasons, Sakha102 had the lowest PW (72.73 and 72.70 g), with a decrease in PL (16.73 and 16.27 cm) and the number of productive tillers (8.67 and 9.33) (**Table 2**). The rice cultivar Giza178 showed a tremendous increase in the PW, PL, and number of productive tillers under drought stress conditions in the two growing seasons tested (**Table 2**).

In addition, drought stress severely reduced the yield and grain yield (GY) plant^-1^ during the reproductive stage in the rice cultivars tested in this study (**Table 3**). Drought stress significantly reduced straw and grain production and the harvest indices (HI) compared with the well-watered control (**Table 3**). Rice cultivars showed significant seasonal variations in GY and its characteristics. The maximum values of PL, PW, and GI were significantly recorded in Giza178 cultivar. On the other hand, Sakha102 produced the lowest GY (**Table 3**). In general, the application of silica increased the values of rice PL, PW, GY, and straw yield (SY) in the two seasons under consideration (**Table 3**). Thus, the highest values were observed at the rate of 400 mg l^-1^ silica.

**Table 3:** Effects of foliar application of silica on the yield in response to drought stress during the growing seasons of 2019 and 2020.

Treatment		Cultivar	GI (g)		GY (g)		SY (g)		HI (%)	
Water status	Silica (mg l^-1^)		2019	2020	2019	2020	2019	2020	2019	2020
**Well-watered (80% WHC)**	**0**	**G178**	31.67 ± 0.64^c^	35.67 ± 0.64^d^	25.64 ± 0.58^f^	26.14 ± 0.58^de^	20.53 ± 0.06^d-g^	21.03 ± 0.06^d-g^	44.48 ± 0.48^a^	44.6 ± 0.47^bc^
		**SK102**	20.00 ± 0.58^h^	20.00 ± 0.58^a^	20.53 ± 0.06^gh^	20.68 ± 0.06^gh^	27.53 ± 0.51^c^	27.68 ± 0.51^c^	42.72 ± 0.64^bcd^	42.77 ± 0.53^bcd^
		**SK107**	25.84 ± 0.03^f^	27.84 ± 0.03^h^	26.59 ± 0.55^de^	26.91 ± 0.55^de^	20.53 ± 0.00^d-g^	20.86 ± 0.00^d-h^	43.59 ± 0.51^ab^	43.67 ± 0.50^a^
	**200**	**G178**	31.67 ± 0.64^c^	35.67 ± 0.64^d^	28.28 ± 0.32^bc^	28.60± 0.32^bc^	20.28 ± 0.08^e-h^	20.61 ± 0.08^e-h^	41.77 ± 0.37^c-f^	41.89 ± 0.36^b^
		**SK102**	22.33 ± 0.06^g^	22.33 ± 0.06^j^	21.34 ± 0.00^g^	21.49 ± 0.00^g^	29.34 ± 0.64^b^	29.49 ± 0.64^b^	42.11 ± 0.64^b-f^	42.17 ± 0.53^b-f^
		**SK107**	27.00 ± 0.29^ef^	29.00 ± 0.29^gh^	27.21 ± 0.00^d^	27.71 ± 0.00^cd^	19.21 ± 0.17^gh^	19.71 ± 0.17^gh^	41.38 ± 0.21^c-g^	41.57 ± 0.21^c-f^
	**400**	**G178**	31.67 ± 0.64^c^	35.34 ± 0.94^de^	30.02 ± 0.03^a^	30.34 ± 0.03^a^	21.51 ± 0.02^de^	21.84 ± 0.03^de^	41.75 ± 0.00^c-f^	41.85 ± 0.00^ab^
		**SK102**	26.67 ± 0.64^f^	26.67 ± 0.64^hi^	22.92 ± 0.07^f^	23.07 ± 0.07^ef^	30.92 ± 0.06^a^	31.07 ± 0.06^a^	42.42 ± 0.78^b-e^	42.61 ± 0.12^bcd^
		**SK107**	29.17 ± 0.64^de^	31.00 ± 0.79^fg^	29.11 ± 0.01^ab^	29.61 ± 0.01^ab^	20.10 ± 0.12^e-h^	20.60 ± 0.12^e-h^	40.85 ± 0.14^efg^	41.03 ± 0.14^c^
**Drought (40% WHC)**	**0**	**G178**	30.00 ± 0.58^cd^	33.00 ± 0.58^ef^	23.19 ± 0.31^f^	23.52 ± 0.31^ef^	15.32 ± 0.00^i^	15.65 ± 0.00^i^	39.79 ± 0.32^g^	39.96 ± 0.31^c-f^
		**SK102**	16.67 ± 0.06^i^	16.67 ± 0.06^k^	18.05 ± 0.58^i^	18.55 ± 0.58^i^	11.05 ± 0.00^j^	11.55 ± 0.00^e-h^	38.00 ± 0.76^h^	38.40 ± 0.74^e-g^
		**SK107**	23.34 ± 0.32^g^	24.84 ± 0.32^i^	19.59 ± 0.00^h^	19.74 ± 0.00^h^	28.33 ± 0.06^bc^	28.48 ± 0.06^bc^	40.66 ± 0.64^fg^	40.94 ± 0.05^bcd^
	**200**	**G178**	40.00 ± 0.58^b^	43.33 ± 0.88^b^	27.44 ± 0.31^cd^	27.77 ± 0.31^cd^	19.94 ± 0.27^fgh^	20.27 ± 0.27^fgh^	42.09 ± 0.61^b-f^	42.20 ± 0.61^b-f^
		**SK102**	20.00 ± 0.58^h^	20.00 ± 0.58^j^	20.86 ± 0.04^g^	21.01 ± 0.04^g^	27.86 ± 0.61^c^	28.01 ± 0.61^c^	42.82 ± 0.64^b-g^	42.87 ± 0.49^bc^
		**SK107**	30.00 ± 0.50^cd^	31.67 ± 0.67^f^	27.02 ± 0.02^d^	27.52 ± 0.02^cd^	19.03 ± 0.58^h^	19.53 ± 0.58^h^	41.30 ± 0.75^c-g^	41.49 ± 0.73^c-f^
	**400**	**G178**	53.99 ± 0.67^a^	57.99 ± 0.67^a^	30.19 ± 0.00^a^	30.52 ± 0.00^a^	21.70 ± 0.32^d^	22.02 ± 0.32^d^	41.81 ± 0.36^c-f^	41.91 ± 0.36^c-f^
		**SK102**	21.67 ± 0.64^gh^	21.67 ± 0.64^j^	22.74 ± 0.00^f^	22.89 ± 0.00^f^	30.73 ± 0.00^a^	30.88 ± 0.00^a^	42.52 ± 0.64^b-e^	42.57 ± 0.00^b-f^
		**SK107**	37.84 ± 0.46^b^	39.84 ± 0.46^c^	29.65 ± 0.00^a^	30.15 ± 0.00^a^	20.65 ± 0.64^def^	21.15 ± 0.64^def^	41.03 ± 0.75^d-g^	41.21 ± 0.73^c-f^

Data are presented as mean ± SE. Within columns, values followed by the same letter are not significantly (P>0.05) different. WHC, water-holding capacity; G178, Giza178; SK102, Sakha102; SK107, Sakha107; GI, weight of 1000 grains or grain index; GY, grain yield; SY, straw yield; HI, harvest index.

### 3.2 Effects of drought stress on physiological and biochemical characteristics of rice plants

Drought stress reduced RWC in the leaves of rice cultivars (**Table 4**). At 80% and 40% WHC, the reduction in RWC (average of the two seasons) was 96.28% and 86.81%, respectively, in Giza178 compared to 80.33% and 72.81% in Sakha102. The RWC of Sakha102 was the most affected cultivar to drought stress among the three rice cultivars (**Table 4**). Similar observations were recorded in the second growing season (**Table 4**).

**Table 4:** Effects of foliar application of silica on RWC and proline content in response to drought stress during the growing seasons of 2019 and 2020.

Treatment		Cultivar	RWC (%)		Pro (μg g FW^-1^)	
Water status	Silica (mg l^-1^)		2019	2020	2019	2020
**Well-atered (80% WHC)**	**0**	**G178**	96.28 ± 0.64^a^	96.63 ± 0.64^a^	1.24 ± 0.01^fg^	1.41 ± 0.01^f^
		**SK102**	68.18 ± 0.04^g^	69.06 ± 0.04^g^	1.14 ± 0.07^g^	1.26 ± 0.07^fg^
		**SK107**	82.23 ± 0.32^c^	82.85 ± 0.32^c^	1.19 ± 0.03^j^	1.33 ± 0.04^i^
	**200**	**G178**	75.65 ± 0.64^e^	76.00 ± 0.64^e^	2.53 ± 0.01^bcd^	2.70 ± 0.01c^d^
		**SK102**	70.10 ± 0.06^fg^	70.98 ± 0.06^fg^	1.85 ± 0.06^def^	1.97 ± 0.06^de^
		**SK107**	72.88 ± 0.34^f^	73.49 ± 0.34^ef^	2.19 ± 0.03^bcd^	2.34 ± 0.03^de^
	**400**	**G178**	78.61 ± 0.61^d^	78.96 ± 0.61^d^	2.84 ± 0.51^b^	3.00 ± 0.51^b^
		**SK102**	81.44 ± 0.06^cd^	82.32 ± 0.06^c^	2.09 ± 0.00^cd^	2.21 ± 0.00^cd^
		**SK107**	80.03 ± 0.29^cd^	80.64 ± 0.29^cd^	2.47 ± 0.25^bcd^	2.61 ± 0.25^c^
**Drought (40% WHC)**	**0**	**G178**	53.55 ± 2.80^i^	53.90 ± 2.80^i^	2.43 ± 0.30^bcd^	2.59 ± 0.30^c^
		**SK102**	37.94 ± 0.09^k^	38.82 ± 0.09^k^	1.37 ± 0.06^efg^	1.49 ± 0.06^ef^
		**SK107**	45.75 ± 1.36^j^	46.36 ± 1.36^j^	1.9 ± 0.12^de^	2.04 ± 0.12^d^
	**200**	**G178**	71.10 ± 0.64^f^	71.45 ± 0.64^fg^	2.76 ± 0.00^bc^	2.92 ± 0.00^bcd^
		**SK102**	39.66 ± 0.06^k^	40.54 ± 0.06^k^	1.92 ± 0.06^efg^	2.04 ± 0.06^ef^
		**SK107**	63.24 ± 0.31^h^	63.85 ± 0.30^h^	2.34 ± 0.03^bcd^	2.48 ± 0.03^cd^
	**400**	**G178**	86.81 ± 0.64^b^	87.16 ± 0.64^b^	4.54 ± 0.06^a^	4.70 ± 0.06^a^
		**SK102**	40.00 ± 0.00^k^	40.88 ±0.00^k^	3.93 ± 0.07^b^	4.05 ± 0.07^a^
		**SK107**	55.55 ± 0.32^i^	56.17 ± 0.32^i^	4.24 ± 0.00^a^	4.38 ± 0.00^a^

Data are presented as mean ± SE. Within columns, values followed by the same letter are not significantly (P>0.05) different. WHC, water-holding capacity; G178, Giza178; SK102, Sakha102; SK107, Sakha107; RWC, relative water content; Pro, proline.

The three tested rice cultivars increased proline content in their leaves when water was deficit (**Table 4**). In the 2019 and 2020 growing seasons, there was an increase of 199.18% and 187.68% in proline content in their leaves, respectively, in Giza178 cultivar plants exposed to drought compared to those that were well-watered. Sakha102, on the other hand, showed the lowest increase (121.20% in 2019 and 124.00% in 2020 more than the well-watered control plants) (**Table 4**).

Photosynthetic pigment contents were altered in leaves of rice plants in response to drought treatment (**Table 5**). Commonly, the leaves showed significant reductions in the total Chl content. Using flood irrigation, Giza178, Sakha102, and Sakha107 had 57.99%, 51.77%, and 56.70% of the total Chl content in the first season and 51.15%, 48.07%, and 49.07% in the second season, respectively (**Table 5**). Drought stress reduced the total Chl content in Giza178 (from 51.70 to 49.99 mg cm^-2^); Sakha102 (from 44.78 to 41.32 mg cm^-2^) and Sakha107 (from 51.92 to 48.32 mg cm^-2^) in the first and second season, respectively (**Table 5**). These findings revealed that Giza178 experienced a modest decrease in the total Chl content when subjected to drought stress. In contrast, Sakha102 was more sensitive to drought. It is worth mentioning that a similar pattern was found in Chl *a* and *b* contents of the three examined rice cultivars in response to well-watered and drought stress treatments in both seasons (**Table 5**).

**Table 5:** Effects of foliar application of silica on photosynthetic pigments in response to drought stress during the growing seasons of 2019 and 2020.

Treatment		Cultivar	Chl *a* (µg cm^-2^)		Chl *b* (µg cm^-2^)		Total Chl (µg cm^-2^)	
Water status	Silica (mg l^-1^)		2019	2020	2019	2020	2019	2020
**Well-watered (80% WHC)**	**0**	**G178**	29.58 ± 0.19^g^	32.45 ± 0.18^ghi^	14.34 ± 0.08^ij^	14.59 ± 0.08^ij^	57.92 ± 0.12^b^	51.45 ± 0.18^cde^
		**SK102**	29.40 ± 0.06^g^	31.3 ± 0.06^i^	14.76 ± 0.64^hij^	13.26 ± 0.64^ij^	51.77 ± 0.06^cd^	51.27 ± 0.06^cde^
		**SK107**	29.49 ± 0.09^fg^	31.88 ± 0.06^hi^	14.75 ± 0.04^hij^	13.93 ± 0.34^ij^	54.85 ± 0.08^bc^	53.15 ± 0.08^c^
	**200**	**G178**	32.33 ± 0.23^fg^	33.51 ± 0.21^fg^	15.49 ± 0.22^ghi^	15.74 ± 0.22^gh^	50.15 ± 0.07^de^	49.33 ± 0.08^e^
		**SK102**	30.38 ± 0.64^g^	32.28 ± 0.64^hi^	16.22 ± 0.06^efg^	14.72 ± 0.06^g^	46.65 ± 0.01^ef^	46.15 ± 0.01^f^
		**SK107**	31.36 ± 0.22^g^	32.89 ± 0.42^gh^	15.68 ± 0.11^fgh^	15.23 ± 0.14^fg^	52.40 ± 3.05^cd^	50.87 ± 1.56^cde^
	**400**	**G178**	35.44 ± 0.26^b^	36.3 ± 0.08^cd^	17.23 ± 0.27^cde^	17.48 ± 0.27^cd^	51.68 ± 0.15^cd^	51.65 ± 0.17^cd^
		**SK102**	32.33 ± 0.07^e^	34.23 ± 0.07^ef^	17.58 ± 0.06^bcd^	16.08 ± 0.06^cd^	52.51 ± 0.64^cd^	52.01 ± 0.64^c^
		**SK107**	33.89 ± 0.13^ef^	35.27 ± 0.04^de^	16.95 ± 0.07^de^	16.78 ± 0.16^c^	51.09 ± 1.04^cd^	51.37 ± 0.58^cde^
**Drought (40% WHC)**	**0**	**G178**	29.79 ± 0.09^f^	28.15 ± 0.02^j^	14.51 ± 0.16^hij^	14.05 ± 0.03^h^	52.49 ± 0.26^cd^	49.41 ± 0.16^de^
		**SK102**	25.13 ± 0.05^e^	27.03 ± 0.05^j^	12.71 ± 0.00^k^	11.21 ± 0.00^k^	44.48 ± 0.06^f^	43.98 ± 0.06^g^
		**SK107**	27.46 ± 0.06^c^	27.59 ± 0.02^j^	13.73 ± 0.03^jk^	12.98 ± 0.08^jk^	38.21 ± 0.02^g^	43.81 ± 0.09^g^
	**200**	**G178**	35.66 ± 0.22^ab^	39.24 ± 0.12^b^	18.65 ± 0.16^b^	18.90 ± 0.16^bc^	52.64 ± 0.07^cd^	58.64 ± 0.19^a^
		**SK102**	29.36 ±0.00^i^	31.26 ± 0.00^e^	17.59 ± 0.39^bcd^	16.09 ± 0.39^cd^	51.78 ± 0.64^cd^	51.28 ± 0.64^cde^
		**SK107**	32.51 ± 0.11^h^	35.25 ± 0.06^de^	16.26 ± 0.05^efg^	17.49 ± 0.18^fg^	52.21 ± 0.29^cd^	55.43 ± 0.24^b^
	**400**	**G178**	36.68 ± 0.08^a^	41.44 ± 0.03^a^	21.63 ± 0.12^a^	21.88 ± 0.12^a^	64.31 ± 0.18^a^	60.19 ± 0.00^a^
		**SK102**	30.55 ± 0.73^g^	32.45 ± 0.73^ghi^	18.33 ± 0.64^bc^	16.83 ± 0.64^cde^	52.36 ± 0.01^cd^	51.86 ± 0.01^c^
		**SK107**	33.62 ± 0.29^e^	36.95 ± 0.37^c^	16.81 ± 0.14^def^	19.35 ± 0.31^bc^	58.33 ± 0.08^b^	59.26 ± 0.04^a^

Data are presented as mean ± SE. Within columns, values followed by the same letter are not significantly (P>0.05) different. WHC, water-holding capacity; G178, Giza178; SK102, Sakha102; SK107, Sakha107; Chl, chlorophyll.

**Figure 1:**
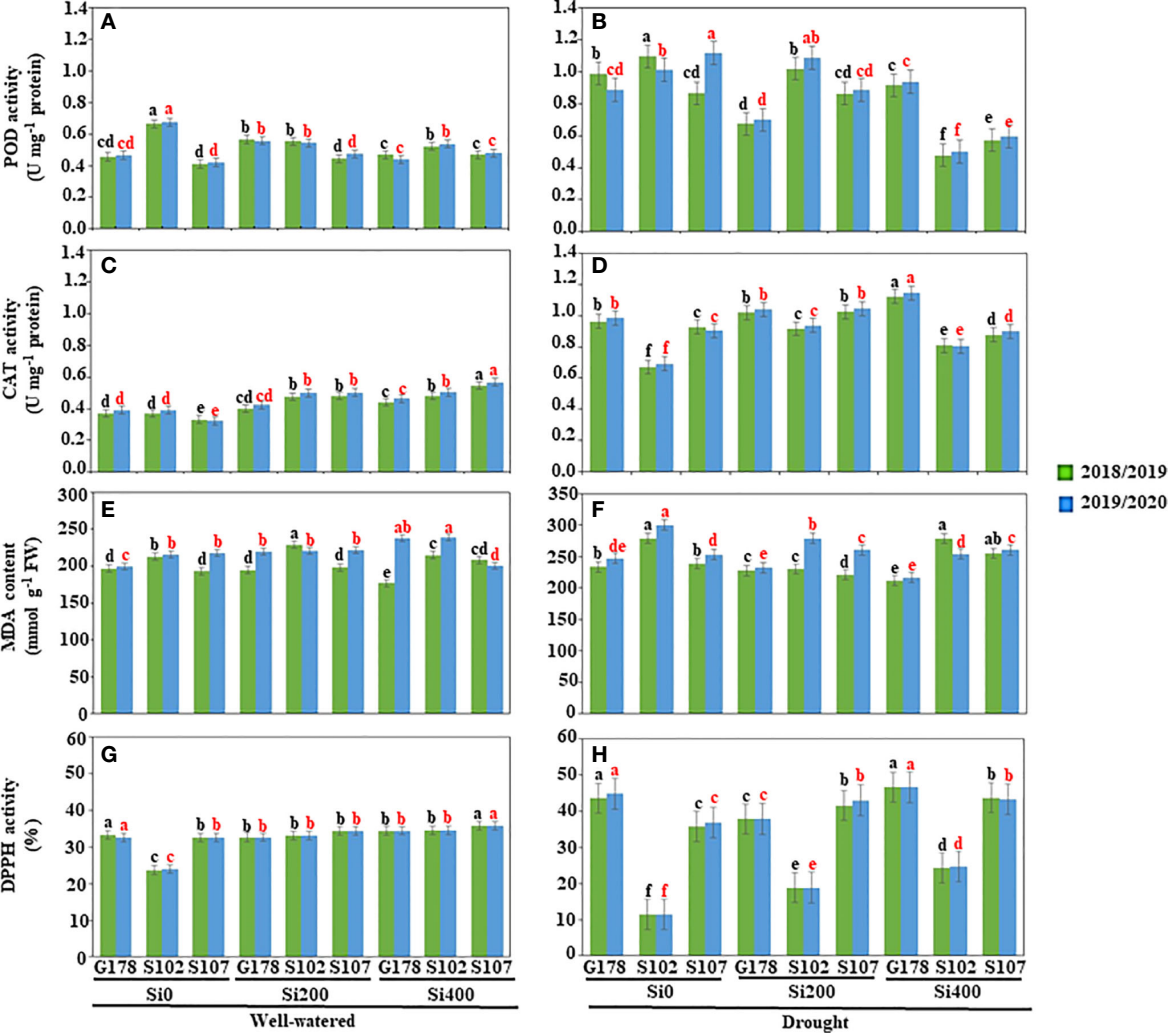
Effects of silica (Si) on enzyme activities, lipid peroxidation, and antioxidant potential of rice under drought conditions. Estimation of the antioxidant enzyme activities of **(A, B)** POD (U mg^–1^ protein) and **(C, D)** CAT (U mg^–1^ protein); measurement of **(E, F)** MDA content (nmol g^-1^ FW); and **(G, H)** DPPH radical scavenging activity (%) under well-watered **(A, C, E, G)** and drought stress **(B, D, F, H)** conditions. Values are means of six replicates ± standard deviation. Different letters in black and red indicated significant differences during the 2018/2019 and 2019/2020 seasons, respectively. POD, peroxidase; CAT, catalase; MDA, malondialdehyde; DPPH, and 2,2-diphenyl-1-picrylhydrazyl.

Furthermore, drought caused activation of the antioxidant system in plant tissues of the three rice cultivars. These findings revealed a significant increase in POD and CAT activities. The enzyme activity of POD, measured at the flowering phase, varied from 0.411 in Sakha107 to 0.665 in Sakha102 under well-irrigated conditions in the first season and from 0.421 to 0.675 in the second season for the same cultivars (**Figure 1A**). When water stress was imposed by withholding water, the POD enzyme activity increased from 0.989 to 1.098 in Giza178 cultivar, and from 0.888 to 1.112 in Sakha102 cultivar (**Figure 1B**). The cultivar Giza178 treated with silica at 400 mg l^-1^ significantly reduced the POD activity in both seasons when drought stress was applied (**Figure 1B**).

The tissue antioxidant enzyme CAT (**Figures 1C, D**) was also checked in the three rice cultivars. In general, the higher the antioxidant enzyme activity during drought stress, the lower the lipid peroxidation degrees the cultivar had. A significant increase in CAT activity was noticed when rice cultivars were supplied with silica. In general, Sakha102 showed a higher CAT activity than Giza178 and Sakha107 in both seasons (**Figures 1C, D**).

The content of MDA was also assessed at the flowering stage for the three rice cultivars under well-watered (**Figure 1E**) and drought (**Figure 1F**) conditions. Except for Giza178, MDA showed increased content levels in stressed plants. However, the lipid peroxidation rate increased regardless of the stage; thus, the maximum increase was recorded in Sakha102 in both seasons (**Figures 1E, F**).

Free radical scavenging in unstressed and stressed rice cultivars was also monitored (**Figures 1G, H**). Our results showed that the antioxidant activities of rice were enhanced under water-stressed conditions, and the antioxidative strength was proportional to drought tolerance levels. DPPH radical scavenging was used as a tool to determine the total antioxidant capacity in rice cultivars. The rapid increase in DPPH radical scavenging capacity could also be related to the level of stress tolerance in plants. The lower the value, the greater the activity. Thus, Sakha102 demonstrated more DPPH radical scavenging activity than Giza178 and Sakha107, suggesting that Sakha102 could be considered as drought-sensitive cultivar (**Figures 1G, H**).

### 3.3 Effect of foliar application of silica on physiological and biochemical characteristics of rice plants

Under drought conditions, silica-treated plants had higher water potential and RWC than those without silica treatment (**Table 4**). Among all cultivars, the best results were obtained with Giza178 with Si400 treatment. All tested cultivars had significant increases in the proline contents as silica was applied on plants whether they were well-watered or drought-stressed (**Table 4**). We also found that the treatment which included 400 mg l^-1^ of silica with Giza178 under drought stress conditions increased the contents of Chl *a* and *b* content compared with those of untreated stressed plants of the same cultivar in both tested seasons (**Table 5**).

The application of silica reduced oxidative damage and enhances drought tolerance in rice plants during the stressful conditions of drought. When compared to well-watered treatments, the activities of POD and CAT in drought-stressed plants without silica application (control) were clearly reduced (**Figures 1A, C**). However, the application of silica increased their activities under drought (**Figures 1B, D**). In response to drought stress, spraying rice plants of Giza178 cultivar with 400 mg l^-1^ of silica slightly increased the activities of POD and CAT. In addition, silica considerably reduced the MDA content in leaves of rice plants that belong to Giza178 cultivar upon the exposure of drought stressed (**Figure 1F**).

### 3.4 Anatomical features of rice roots and stems

**Figure 2:**
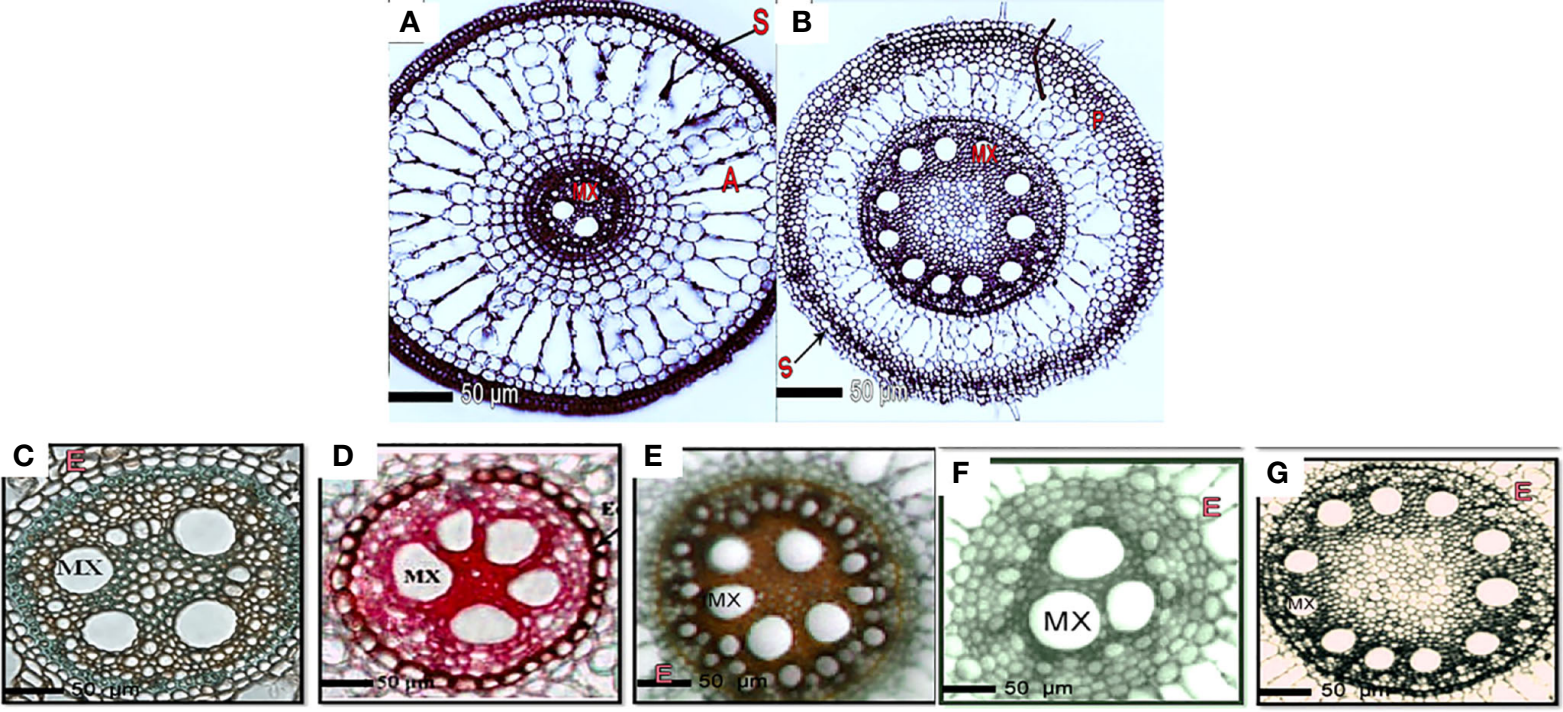
Transverse sections of the formation of root layers in rice cultivars affected by the application of silica and drought stress. Layer formation of aerenchyma, parenchyma, endodermis, and sclerenchyma in Giza178 cultivar in response to **(A)** well-watered (control); **(B)** drought stress for 4 months. Giza178, Sakha102, and Sakha107 were treated with or without silica under drought stress conditions. Response of the drought-tolerant Giza178 cultivar to no silica treatment under **(C)** well-watered irrigation (control) and **(D)** drought stress conditions. Response of the **(E)** moderate tolerant cultivar Sakha107 and **(F)** drought-sensitive Sakha102 cultivars to no silica treatment and drought stress. Alternations in root system architecture of Giza178 upon the application of silica at 400 mg l^-1^ under drought stress conditions. In **(A, B)**, cross sections were obtained at 20 mm from the root tip. In **(C–G)**, roots were stained with safranin and fast green. Notice the cell wall thickness of pith and xylem under drought conditions. E, endodermis; MX, metaxylem.

Under well-irrigated and drought conditions, anatomical features of transverse sections of rice roots clearly distinguished the tolerant and sensitive rice cultivars. In general, rice roots consisted of more aerenchyma and air spaces when plants were well-irrigated than the drought-stressed plants (**Figure 2A**). We observed increase in the size and number of aerenchymatous tissues as well as in secondary cell walls (**Figure 2A**). In response to drought stress, the rice roots in the three cultivars showed a proportional decrease in the cortex area destined for the aerenchyma, in addition to thickening of the cell walls of the endodermis and sclerenchyma layer cells (**Figure 2A**).

The outer layers were made up of unicellular epiblema, followed by the cortex region, which was made up of multilayered cortical parenchymatous tissue and vascular bundles of xylem and phloem tissues. In drought-tolerant cultivars, the rate of aerenchyma disappeared in drought-stressed plants, indicating different morphological alternations in the tested cultivars. Giza178 and Sakha107 showed a significant reduction in aerenchyma tissue when plants were under drought stress. The shortage of water resulted in more sclerenchyma layers in the cell walls in the roots of the 4-month-old rice plants in Giza178 or Sakha107 cultivars. The thicknesses of xylem vessels (XV) under well-watered (control) and drought conditions were 4.6 and 8.9 μm, respectively, in Giza178 cultivar (**Table S4**). In Giza178 cultivar, the thicknesses of the endodermis cell wall in roots ranged from 4.6 under well-irrigated water regimes to 10.8 μm under drought stress conditions, whereas Sakha102 cultivar showed less thicknesses in the endodermis cell wall (**Figure 2B**). In general, drought stress also affected the lignification process in the epidermis (ET), exodermis, and sclerenchyma of roots (**Figure 2B**).

Under control and drought conditions, the anatomical features of transverse rice roots differentiated tolerant and sensitive cultivars (**Table S4**). In comparison to the root system of well-watered plants (**Figure 2C**), the stele diameter increased and was positively connected with the XV area under drought stress conditions (**Figures 2D–F**). Thus, this was essential for the water conductance from the soil to the top regions of the plants to satisfy evaporative need. In response to drought stress, the vascular cylinder area was affected differently in the three rice varieties. For example, the area of the vascular cylinder was highly affected in Sakha107 (**Figure 2E**), whereas the same area continued to expand in Giza178 (**Figure 2D**) by drought stress.

When plants of Giza178 cultivar were exposed to water stress, Casparian strips known for the endodermis suberization in roots were primarily made of suberin, a waxy substance surrounding the endodermis that could potentially help the root system resist water (**Figure 2D**). In addition, we observed that the expansion in the vascular cylinder area in the roots of Giza178 cultivar increased metaxylem (MX) vessel numbers in the small-diameter vessels in response to drought (**Figure 2C**) compared to well-irrigated treatments (**Figure 2D**). In Sakha102, the area of the vascular cylinder was limited when drought stress was applied on these plants (**Figure 2F**). Thus, this susceptible variety had the least amount of MX within the roots.

On the other hand, the increased levels of silica in combination with the drought-tolerant cultivar Giza178 resulted in a significant increase in the diameter of the vascular cylinder of roots even under drought stress conditions (**Figure 2G**). The root of the examined cultivars produced thicker cell walls in the endodermis, xylem vessels, and sclerenchyma layer cells in response to the drought conditions; thus, this response was noticed more frequently in Giza178 cultivar. This suggests that the Giza178 cultivar may most probably be more tolerant to drought stress than Sakha107 or Sakha102 cultivars (**Figure 2**). In conclusion, this study demonstrated that the root anatomical features of the cultivar Giza178 seemed to have a better response to tolerate drought stress when compared to other rice cultivars, i.e., Sakha102.

In the current study, well-irrigated and water-deficient treatments reduced most of the Sakha102 cultivar stem anatomical features compared with the control (**Table S5** and **Figure S1**). A significant reduction in ET, phloem tissue (PhT), and inner vascular bundle thickness (VBT; µm) was observed. However, it was clear from the results that all stem anatomical features of the rice cultivar Giza178 increased significantly in response to drought stress compared to well-watered control plants (**Table S5**).

In comparison to plants that were or were not exposed to drought stress, the application of silica at 400 mg l^-1^ increased the diameter of the metaxylem vessels and the thickness of the stem ground tissue (**Table S5**).

### 3.5 Economic indicators of land and yield components

Food security is necessary for the economic and social stability as well as for sustainable development. In Egypt, the rice crop is of further importance to farmers for profitable purposes. Because it is grown mainly in the Nile River Delta (e.g., the city of Kafr Sheikh), the rice growing areas face common water shortage during the production season. Such area can be used for rice cultivation to alleviate the effect of salinity stress. One effective strategy to overcome such problem is to develop improved varieties with a better genetic composition for low water consumption.

Drought stress had a negative impact on yield-contributing traits, such as PW, PL, and the number of productive tillers. In both seasons, Sakha102 had the lowest PW, and PL and the number of productive tillers were reduced. Under drought stress conditions, the rice cultivar G178 showed a tremendous increase in these traits in both seasons. In addition, SY and GY were severely reduced in the tested rice genotypes under drought stress conditions (**Table 3**).

The various improvement techniques of rice varieties play a pivotal role in increasing productivity per unit area of land and in the amount of water used to produce. The average annual land and water productivity of rice varieties were analyzed in this study. The Earth’s average productivity for these varieties was estimated at 9.58 MT ha^-1^. The two cultivars, Giza178 and Sakha102, recorded the highest and lowest average production ha^-1^, respectively. These results indicated that Giza178 presents a 2% higher productivity compared to the average production of the other varieties. This is equivalent to an increase in rice production of 31,000 MT year^-1^, which can be due to its high production capacity with a shorter maturity duration compared to other rice cultivars. According to the current findings, the cultivar Giza178 was found to be drought tolerant, producing higher GY and SY than all other cultivars tested under drought stress conditions. However, Sakha102, the drought-prone cultivar, had the lowest GY and SY (**Table 3**).

### 3.6 Correlation analysis

**Figure 3:**
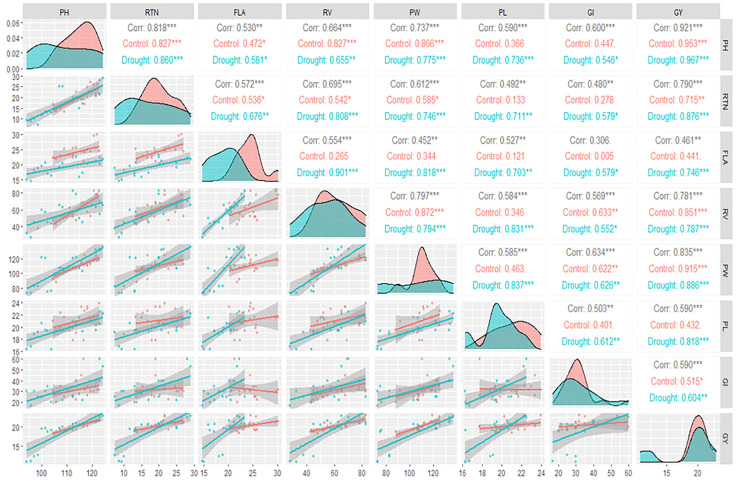
Spearman correlation matrix among the studied traits under well-watered (control) and drought treatments. The diagonal part of the produced plot represents the density plots of the studied traits, while the lower triangle represents the regression relationship with confidence interval between each pair of the studied traits. *, ** and *** refer to significant difference at P<0.01, 0.05 and 0.001, respectively.

The upper triangle revealed that GY was significantly correlated with all other traits under control and drought treatments except for PL and FLA under control treatment only (**Figure 3**). In addition, the diagonal showed the density plots of the investigated traits (**Figure 3**). The X-axis of each density plot represents the values of the trait, while the Y-axis represents the relative probability of an area under the curve (**Figure 3**).

The highest density of the values of the trait is referred by the area under the curve around the peak of the density plot. From the density plots, it was observed that all the studied traits were affected by the drought treatment mainly in its density as well as its magnitude, where the peaks of the studied traits under drought treatment (blue plots) were at lower values than the peaks under control treatment (red plots) (**Figure 3**).

### 3.7 Path analysis

**Figure 4:**
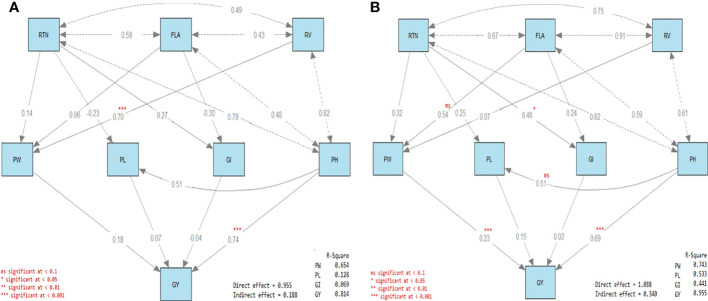
Path diagram of the direct and indirect effects of water irrigation regimes on rice traits. Response traits in rice plants under **(A)** well-watered (control) and **(B)** drought stress conditions. Each diagram has three types of arrows. The first is the path of a single-headed arrow that is used to define the causal relationships between two variables, where the variable at the tail of the arrow (independent variable) affects the variable at the head (dependent variable). The second is the covariance, which is a double-headed arrow connecting two variables and defining the covariance between them. The third is the variance, which is a double-headed arrow pointed at the same variable and defining the variance of that variable.

The four direct effects and the three indirect effects are included in **Figure 4**. Concerning the direct effects, the first was the direct effect of reproductive tiller number (RTN), FLA, and RV on PW (R^2^ = 0.654 and 0.743 under control and drought treatments, respectively; **Figure 4A**). The second was the direct effect of RTN and FLA on GI (R^2^ = 0.069 and 0.441 under control and drought treatments, respectively). The third was the effect of RTN and PH on PL (R^2^ = 0.128 and 0.533 under control and drought treatments, respectively). The fourth was the direct effect of PW, PL, GI, and PH on GY (R^2^ = 0.814 and 0.955 under control and drought treatments, respectively).

On the other hand, the first was the indirect effect of RTN, FLA, and RV on GY *via* PW, and the second was the indirect effect of RTN and PH on GY *via* PL (**Figure 4B**). The third was indirect effect of RTN and FLA on GY was *via* GI. The significant direct effects were RV on PW and PH on GY under control treatment, while under drought treatment, the significant direct effects were RTN on GI and both PW and PH on GY. All the indirect effects were not significant either under control or under drought treatment (**Figure 4**).

**Figure 5:**
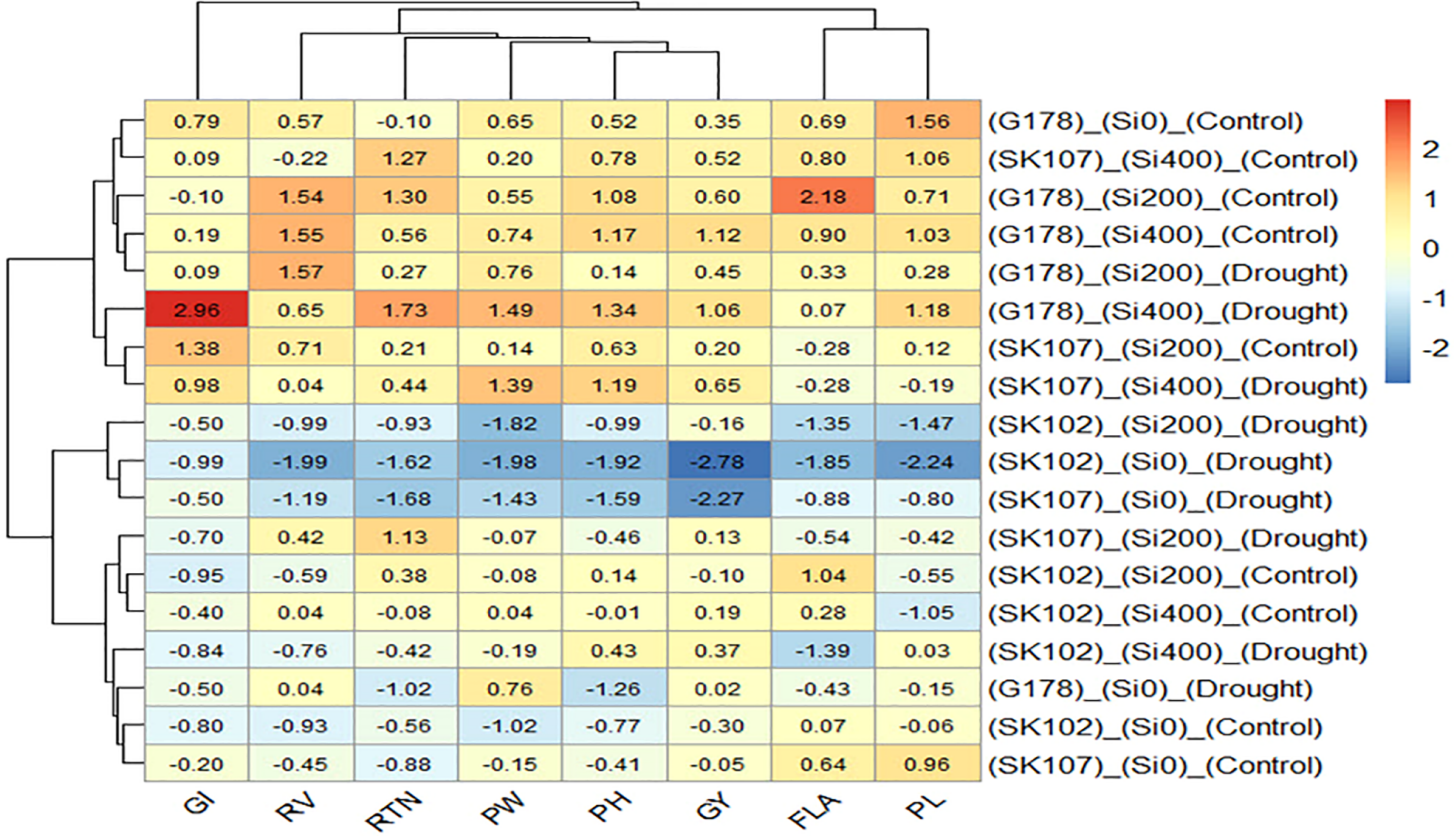
Heatmap of the relationship among the treatments and the studied traits. Cells with red color represents elevated values, while cells with blue color represents reduced values of the traits. G178, Giza178; SK102, Sakha102; SK107, Sakha107; GI, grain index; RV, root volume; RTN, reproductive tiller number; PW, panicle weight; HI, harvest index; GY, grain yield; FLA, flag leaf area; PL, panicle length.

From the heatmap, it was clear that the lower GY (blue color) was mainly due to the lower values of all the traits except for GI (**Figure 5**). In addition, the higher GY (red color) was associated with the high values of all traits except for GI (**Figure 5**).

### 3.8 The goodness-of-fit model

As the characteristics of the goodness of fit were as shown in **Table 6**, almost all indices meet the criteria such as the ratio of chi-square to degrees of freedom (CMIN/df) = 0.003/11 (<2). It was demonstrated that the normed fit index (NFI) = 0.987, comparative fit index (CFI) = 0.989, Tucker–Lewis index (TLI) = 0.978, and relative fit index (RFI) = 0.973.

**Table 6:** Goodness-of-fit measures for model evaluation.

Goodness-of-fit index	Indicator value	Critical (acceptable) value	Status
**CMIN/df**	0.003/11	<2	Goodness of fit
**NFI**	0.987	>0.9	Goodness of fit
**CFI**	0.989	>0.9	Goodness of fit
**TLI**	0.978	>0.9	Goodness of fit
**RFI**	0.973	>0.9	Goodness of fit
**GFI**	0.975	>0.9	Goodness of fit
**RMSEA**	0.004	<0.05	Goodness of fit

CMIN/df, chi-square to degrees of freedom; NFI, normed fit index; CFI, comparative fit index; TLI, Tucker–Lewis index; RFI, relative fit index; GFI, goodness-of-fit index; RMSEA, root mean square error of approximation.

Another way to fit this model was to calculate the approximate high goodness-of-fit index (GFI) = 0.975, and the estimated root mean square error of approximation (RMSEA) = 0.004 (<0.05), confirming the fitness of the model and representing the best fit to the model.

## 4 Discussion

Drought stress is one of the significant environmental stress factors affecting plant growth and development (Singh et al., 2017). The lack of soil moisture may partially contribute to the negative impact on plant growth and development (Gong et al., 2003; Singh et al., 2017; Abd El-Mageed et al., 2022). This was evidenced by the reduction in all growth parameters reported in the current study. In alignment with other studies (Henry et al., 2016; Elnahal et al., 2022; Fouda et al., 2022), our results showed that water stress reduced PH and the number of tillers. This could be due to the decrease in cell turgor, which inhibited cell division and expansion. The PH and number of tillers varied among the three rice cultivars tested in the present study.

Leaf area is a critical factor that influences crop development and production and is primarily responsible for the plant’s photosynthetic activity. The decline in FLA in our study might have resulted from the compact size and senescence of leaves, as well as the short growing season (Zewdie et al., 2007; Prasad et al., 2021; Naiem et al., 2022a; Naiem et al., 2022b). It was found that Giza178 was the least influenced rice cultivars tested in this study under drought stress conditions in both seasons (**Table 1**). Thus, Giza178 was considered to be a drought-tolerant cultivar producing the highest GY and SY under drought stress conditions among the tested cultivars, whilst Sakha102 yielded the least grains and straws, and it was regarded as drought-sensitive cultivar.

During the reproductive stage of rice, the increase in soil–water tension increased spikelet abortion, resulting in a reduction in the spikelet counts panicle^-1^ (Kikuta et al., 2016). In addition, the decrease in GY decreased the FLA and photosynthetic ratio (Kumar and Dey, 2011; Lemoine et al., 2013). In the current study, GY was proportionally reduced in response to the water-deficit treatment in both seasons (**Table 2**) and this could be because of the shortage in water supply. In the current investigation, low GY was associated with low GI.

According to Ahmed et al. (2011), drought tolerance in plants can be enhanced by the application of silica. When plants are exposed to drought stress, their leaf water potential and RWC decreases (Farooq et al., 2009). Therefore, silica application can improve water status in rice and other crops under drought conditions (Farooq et al., 2009; Gong and Chen, 2012). Similar observations have been reported by Sonobe et al. (2011) in sorghum plants treated with silica under water-deficit stress conditions. It has been proposed that incorporating silica into the culture solution may improve root water uptake by the root system under drought stress by the active deposition of total soluble sugars and amino acids.

To reverse the harmful effects of drought injuries, plants can maintain cell turgor by accumulating different types of organic and inorganic solutes (sugars, amides, amino acids, and proline) in the cytosol (Joseph et al., 2015). The increased proline content can help maintain the tissue water status and prevent cell damage caused by the drought. This is consistent with the results reported by Huang et al. (2004) who demonstrated that low water potential can cause cell membrane destruction and enzyme deactivation, thereby leading to electrolyte loss. Proline deposition takes place typically in the cytosol, resulting in cytoplasmic osmotic adjustment. In the current study, an elevation in proline contents was shown in drought-stressed rice plants. Giza178 accumulated more proline in response to drought stress than any other rice cultivar (**Table 4**). This could be attributed to its high efficiency in drought tolerance. This is in agreement with the reported findings of Khedr et al. (2022), when they showed that the rice cultivar Misr 3 exposed to high salt stress resulted in higher proline content, compared to plants not suffering from salt stress.

Proline levels increased when wheat leaves were exposed to water stress, whereas silica application reduces accumulation of proline (Khedr et al., 2022). We argue that the accumulation of proline can be an indicator of drought stress-related damage. More research is required to understand the role of the regulative role of silica in the accumulation of compatible cellular solutes in drought tolerance.

One strategy to ameliorate the detrimental effects of oxidative stress through the overaccumulation of reactive oxygen species (ROS) in drought-tolerant plants is the generation of both enzymatic and non-enzymatic antioxidant defense systems under harsh environmental conditions (Cui et al., 2010). Our data in the three tested cultivars showed an elevation in antioxidant capacity in response to drought, of which Giza178 had the highest and Sakha102 the lowest values. These data were in agreement with those previously reported by Dominguez-Perles et al. (2011).

It has been reported that water deficiency also alters the biochemical responses in plants by increasing the antioxidant capacity (Anjum et al., 2012; Lum et al., 2014; Abd El-Aty et al., 2022; Abo Sen et al., 2022). For instance, drought stress can enhance the activities of the antioxidant enzymes, superoxide dismutase (SOD), CAT, and POD, thus developing defense mechanisms against ROS (Khedr et al., 2022). SOD catalyzes the dismutation of superoxide radicals to molecular oxygen and H_2_O_2_, providing cellular defense against ROS. The majority of the H_2_O_2_ produced by the catalysis of SOD remained biologically toxic. Following drought stress, the increased rate of SOD activity was lower in drought-tolerant genotypes than in drought-sensitive genotypes; however, the increased rate of CAT and POD activity and total antioxidant capacity was higher in the drought-tolerant genotypes (Simova-Stoilova et al., 2008; Hussain et al., 2021; El-Ashry et al., 2022; Khedr et al., 2022). Thus, the production of H_2_O_2_ due to the activated SOD enzyme can function in oxidative stress signaling and can act as a secondary messenger to protect reactions leading to induced CAT and POD activity in plants (Anjum et al., 2011; Hussain et al., 2021; Abd El-Mageed et al., 2022; El-Ashry et al., 2022). The drought-induced oxidative stress tolerance may be conferred by the high stability and increased CAT and POD activities (Tian et al., 2012).

MDA which is produced by membrane lipids in response to ROS can be used as a drought indicator to evaluate the degree of plasma membrane damage and the ability of plants to tolerate drought stress (Zhang et al., 2021). The high contents of free proline and MDA were also associated with rice productivity under drought stress conditions, despite the cultivar used.

Silica mitigates drought stress in many plants, including rice, wheat, maize, tomato, sorghum, sugarcane and broad bean, which is largely due to improved water retention and photosynthesis (Gong and Chen, 2012; Malik et al., 2021). In wheat, silica partially reduced the detrimental effect of drought by enhancing the activities of SOD, CAT, and glutathione reductase while decreasing the H_2_O_2_ concentration and oxidative protein destruction (Gong et al., 2005). In addition, the activity of acid phospholipase, which hydrolyzes phospholipids, was reduced in wheat plants treated with silica and exposed to drought stress, thus indicating that silica can lower the phospholipid de-esterification damage in drought-stressed wheat. In grapevine rootstock, silica reduces the MDA content (Soylemezoglu et al., 2009). DPPH free radicals were also investigated for screening plant cultivars for stress tolerance (Dominguez-Perles et al., 2011). The increase in the DPPH radical scavenging capacity can be correlated with the degree of stress tolerance in plants (Huang et al., 2004; Joseph et al., 2015).

Lignification and suberization of plant structures can also help in maintaining deep soil horizon water uptake while avoiding losses to dry soil at shallower levels. It has been reported that silica is involved in the formation of Casparian strip in the root endodermis and exodermis and increased suberization and lignification of sclerenchyma in rice cells (Fleck et al., 2011). These characteristics warrant further study before they can be used in agricultural settings. Drought increased the lignification of the stele while decreasing the lignification of the cortex and outer layers (ET, exodermis, and sclerenchyma) in roots. In rice roots, the distribution and reactivity of suberized and lignified endodermal and outer cell layers in wet and dry soils play important roles in controlling water and nutrient homeostasis (Barberon, 2017). Their significance in establishing a barrier to radial oxygen loss during floods has been well documented (Colmar, 2003). Despite the significant reduction in root permeability of rice to drought, the increased lignification and suberization of the exodermis and endodermis have led to the formation of a barrier to radial O_2_ loss which did not affect root hydraulic conductivity (Garthwaite et al., 2006; Ranathunge et al., 2011).

In contrast to sensitive varieties, vascular bundles of drought tolerant cultivars were found to be highly responsive to water deficiency. For example, the vascular bundle diameter has contributed to the maintenance and transport of water (Kadam et al., 2015). In order to preserve stele area under drought stress, it is advantageous to retain root penetration (Kikuta et al., 2016). In the current study, the drought tolerant cultivar Giza178 had a total of five metaxylem vessels; whilst Sakha102, which is far more sensitive, possessed only three vessels. Similar pattern was observed in the number of xylem tissues. As a result, the diameter and quantity of xylem along the root length of tolerant rice types increase water efficiency under water scarcity stress (Kadam et al., 2015). The structures in the root metaxylem can be considered as morphological characteristics in drought stressed plants (Kadam et al., 2015).

Conservation of the stele area under drought stress can be advantageous for maintaining root penetration ability (Kikuta et al., 2016). Smaller vessels can protect the xylem from cavitation, moderate water movement to the shoot, and maintain the rhizosphere wetness for roots to continue growth and water and nutrient uptake. Plasticity in vessel size may provide advantages under drought, while allowing sufficient water transport to the shoot to support growth under well-irrigated conditions. In corn, the stele area was positively associated with root tensile strength (Delavar et al., 2017). In rice, the maintenance of the stele area and fortification of the stele with lignin during drought can help roots to continue growing when soils become harder. In drought tolerant cultivars, the lignification of epidermal tissues and the thickness of vascular and dermal tissues increase in stems of plants under water stress conditions (Dolatabadian et al., 2011). It has also been reported that silica can be involved in the formation of Casparian strips in the root endodermis and exodermis; in addition to the increased suberization and lignification of sclerenchyma tissues in rice plants exposed to drought stress (Fleck et al., 2011). In the present study, the insignificant correlation between GY, PL, and FLA was due to that PL and FLA were not significantly correlated with all the other traits. In general, the correlation coefficients among the studied traits under drought treatment tended to be higher than under control treatment with exception to the correlation between RV and both GY, GI, and PH.

In our path analysis, the direct effect of RV on PW was significant under control treatment; however, it was not significant under drought treatment. This may be due to the adverse effect of drought on root growth. The heatmap showed that Giza178 was superior to Sakha102 and Sakha107 in PH, PW, RTN, GI, and GY. This suggests that GY and its attributes were less adversely impacted by drought than the other cultivars.

## 5 Conclusion

From the results, we conclude that drought stress negatively affects the morphological, physiological, biochemical characteristics of the tested rice cultivars. Silica could promote growth and development in rice by increasing water interactions and enhancing physiological properties. Our results showed that the application of silica on rice plants can not only ameliorate the impact of drought stress but also improve the quality features of the grain. In conclusion, Giza178 was the most drought tolerant, whereas Sakha102 was the most drought sensitive among the tested rice cultivars in this study. Future direction to determine the main biomarkers of drought stress and how these are mitigated by the exogenous application of silica is underway.

## Data availability statement

The original contributions presented in the study are included in the article/**Supplementary Material**. Further inquiries can be directed to the corresponding authors.

## Author contributions

SE-O, ME-A, KE-T, and SA conceived and designed the research. SE-O, ME-A, KE-T, SA, and DS supervised the study. SE-O, SS, AE-T, and OI performed field experiments. SE-O, ME-A, SS, AE-T, and OI developed the biochemical and physiological analyses. ME-S, HA, SA, KE-T, and DS analyzed the data. MN, MA, HA, and ME-S assisted with experiments and/or data evaluation. SE-O, SA, KE-T, and DS wrote the manuscript. All authors contributed to the article and approved the submitted version.

## Funding

This project was funded by the Abu Dhabi Research Award (AARE2019) for Research Excellence-Department of Education and Knowledge (ADEK; Grant #: 21S105) to KE-T and the Khalifa Center for Biotechnology and Genetic Engineering-UAEU [grant number 31R286] to SA.

## Acknowledgments

KE-T would like to thank the library at Murdoch University, Australia, for the valuable online resources and comprehensive databases.

## Conflict of interest

The authors declare that the research was conducted in the absence of any commercial or financial relationships that could be construed as a potential conflict of interest.

## Publisher’s note

All claims expressed in this article are solely those of the authors and do not necessarily represent those of their affiliated organizations, or those of the publisher, the editors and the reviewers. Any product that may be evaluated in this article, or claim that may be made by its manufacturer, is not guaranteed or endorsed by the publisher.

## References

[B1] Abd El-Aty M. S. Abo-Youssef M. I. Galal A. A. Salama A. M. Salama A. A. El-Shehawi A. M. . (2022). Genetic behavior of earliness and yield traits of some rice (*Oryza sativa* l.) genotypes. Saudi J. Biol. Sci. 29, 2691–2697. doi: 10.1016/j.sjbs.2021.12.054 35531209 PMC9072890

[B2] Abd El-Mageed T. A. Rady M. O. A. Abd El-Wahed M. H. Abd El-Mageed S. A. Omran W. M. Aljuaid B. S. . (2022). Consecutive seasonal effect on yield and water productivity of drip deficit irrigated sorghum in saline soils. Saudi J. Biol. Sci. 29, 2683–2690. doi: 10.1016/j.sjbs.2021.12.045 35531259 PMC9073043

[B3] Abo Sen E. Z. F. El-Dahan M. A. A. Badawy S. A. Katta Y. S. Aljuaid B. S. El-Shehawi A. M. . (2022). Evaluation of genetic behavior of some egyption cotton genotypes for tolerance to water stress conditions. Saudi J. Biol. Sci. 29, 1611–1617. doi: 10.1016/j.sjbs.2021.11.001 35280572 PMC8913392

[B4] Aebi H. Wyss S. R. Scherz B. Skvaril F. (1974). Heterogeneity of erythrocyte catalase II. isolation and characterization of normal and variant erythrocyte catalase and their subunits. Eur. J. Biochem. 48, 137–145. doi: 10.1111/j.1432-1033.1974.tb03751.x 4141308

[B5] Ahmad F. Lah M. R. Aziz T. Maqsood M. A. Tahir M. A. Kanwal S. (2007). Effect of silicon application on wheat (*Triticum aestivum* l.) growth under water deficiency stress. Emir. J. Food Agric. 19, 1–7. doi: 10.9755/ejfa.v12i1.5170

[B6] Ahmed M. Hassen F. U. Qadeer U. Aslam M. A. (2011). Silicon application and drought tolerance mechanism of sorghum. Afr. J. Agric. Res. 6, 594–607. doi: 10.5897/AJAR10.626

[B7] Anjum S. A. Farooq M. Xie X. Y. Liu X. J. Ijaz M. F. (2012). Antioxidant defense system and proline accumulation enables hot pepper to perform better under drought. Sci. Hortic. 140, 66–73. doi: 10.1016/j.scienta.2012.03.028

[B8] Anjum S. A. Xie X. Y. Wang L. C. Saleem M. F. Man C. Lei W. (2011). Morphological, physiological and biochemical responses of plants to drought stress. Afr. J. Agric. Res. 6, 2026–2032. doi: 10.5897/AJAR10.027

[B9] Arnon I. (1972). Crop production in dry regions vol. I: Background andPrinciples (London: Leonard Hill), 670 pp.

[B10] Ashraf M. Harris P. J. C. (2013). Photosynthesis under stressful environments: An overview. Photosynthetica 51, 163–190. doi: 10.1007/s11099-013-0021-6

[B11] Barberon M. (2017). The endodermis as a checkpoint for nutrients. New Phytol. 213, 1604–1610. doi: 10.1111/nph.14140 27551946

[B12] Bates L. S. Waldren R. P. Teare I. D. (1973). Rapid determination of free proline for water-stress studies. Plant Soil 39, 205–207. doi: 10.1007/BF00018060

[B13] Bradford M. M. (1976). A rapid and sensitive method for the quantitation of microgram quantities of protein utilizing the principle of protein-dye binding. Anal. Biochem. 72, 248–254. doi: 10.1006/abio.1976.9999 942051

[B14] Chen Y. E. Liu W. J. Su Y. Q. Cui J. M. Zhang Z. W. Yuan M. . (2016). Different response of photosystem II to short and long term drought stress in *Arabidopsis thaliana* . Physiol. Plant. 158, 225–235. doi: 10.1111/ppl.12438 26918860

[B15] Choudhary M. K. Basu D. Datta A. Chakraborty N. Chakraborty S. (2009). Dehydration-responsive nuclear proteome of rice (*Oryza sativa* l.) illustrates protein network, novel regulators of cellular adaptation, and evolutionary perspective. Mol. Cell Proteomics 8, 1579–1598. doi: 10.1074/mcp.M800601-MCP200 19321431 PMC2709188

[B16] Colmar T. D. (2003). Long-distance transport of gases in plants: A perspective on internal aeration and radial oxygen loss from roots. Plant Cell Environ. 26, 17–36. doi: 10.1046/j.1365-3040.2003.00846.x

[B17] Cui X. H. Murthy H. N. Wu C. H. Paek K. Y. (2010). Sucrose-induced osmotic stress affects biomass, metabolite, and antioxidant levels in root suspension cultures of *Hypericum perforatum* l. Plant Cell Tiss. Organ Cult. 103, 7–14. doi: 10.1007/s11240-010-9747-z

[B18] Delavar K. Ghanati F. Zare Maivan H. Behmanesh M. (2017). Effects of silicon on the growth of maize seedlings under normal, aluminum, and salinity stress conditions. J. Plant Nutr. 40, 1475–1484. doi: 10.1080/01904167.2016.1269344

[B19] Dolatabadian A. Sanavy S.A.M. Ghanati F. (2011). Effect of salinity on growth, xylem structure and anatomical characteristics of soybean. Not. Sci. Biol. 3, 41–45. doi: 10.15835/nsb315627

[B20] Dominguez-Perles R. Martinez-Ballesta M. C. Riquelme F. Carvajal M. Garcia-Viguera C. Moreno D. A. (2011). Novel varieties of broccoli for optimal bioactive components under saline stress. J. Sci. Food Agric. 91, 1638–1647. doi: 10.1002/jsfa.4360 21445869

[B21] El-Ashry R. M. El-Saadony M. T. El-Sobki A. E. A. El-Tahan A. M. Al-Otaibi S. El-Shehawi A. M. . (2022). Biological silicon nanoparticles maximize the efficiency of nematicides against biotic stress induced by *Meloidogyne incognita* in eggplant. Saudi J. Biol. Sci. 29, 920–932. doi: 10.1016/j.sjbs.2021.10.013 35197760 PMC8848026

[B22] Elnahal A. S. M. El-Saadony M. T. Saad A. M. Desoky E.-S. M. El-Tahan A. M. Rady M. M. . (2022). The use of microbial inoculants for biological control, plant growth promotion, and sustainable agriculture: A review. Eur. J. Plant Pathol. 162, 759–792. doi: 10.1007/s10658-021-02393-7

[B23] FAOSTAT (2017). “Food and agriculture organization of the united nations,” in FAOSTAT statistical database (Rome: FAO).

[B24] Farooq M. Basra S. M. A. Wahid A. Cheema Z. A. Cheema M. A. Khaliq A. (2008). Physiological role of exogenously applied glycinebetaine to improve drought tolerance in fine grain aromatic rice (*Oryza sativa* l.). J. Agron. Crop Sci. 194, 325–333. doi: 10.1111/j.1439-037X.2008.00323.x

[B25] Farooq M. Wahid A. Kobayashi N. Fujita D. Basra S. M. A. (2009). Plant drought stress: Effects, mechanisms and management. Agron. Sustain. Dev. 29, 185–212. doi: 10.1051/agro:2008021

[B26] Fleck A. T. Nye T. Repenning C. Stahl F. Zahn M. Schenk M. K. (2011). Silicon enhances suberization and lignification in roots of rice (*Oryza sativa*). J. Exp. Bot. 62, 2001–2011. doi: 10.1093/jxb/erq392 21172812 PMC3060683

[B27] Fouda S. E. E. El-Saadony F. M. A. Saad A. M. Sayed S. M. El-Sharnouby M. El-Tahan A. M. . (2022). Improving growth and productivity of faba bean (*Vicia faba* l.) using chitosan, tryptophan, and potassium silicate anti-transpirants under different irrigation regimes. Saudi J. Biol. Sci. 29, 955–962. doi: 10.1016/j.sjbs.2021.10.007 35197763 PMC8847969

[B28] Garthwaite A. J. Steudle E. Colmer T. D. (2006). Water uptake by roots of *Hordeum marinum*: Formation of a barrier to radial O_2_ loss does not affect root hydraulic conductivity. J. Exp. Bot. 57, 655–664. doi: 10.1093/jxb/erj055 16410258

[B29] Gong H. Chen K. (2012). The regulatory role of silicon on water relations, photosynthetic gas exchange, and carboxylation activities of wheat leaves in field drought conditions. Acta Physiol. Plant. 34, 1589–1594. doi: 10.1007/s11738-012-0954-6

[B30] Gong H. J. Chen K. M. Chen G. C. Wang S. M. Zhang C. L. (2003). Effects of silicon on growth of wheat under drought. J. Plant Nutr. 26, 1055–1063. doi: 10.1081/PLN-120020075

[B31] Gong H. J. Chen K. M. Zhao Z. G. Chen G. C. Zhou W. J. (2008). Effects of silicon on defense of wheat against oxidative stress under drought at different developmental stages. Biol. Plant 52, 592–596. doi: 10.1007/s10535-008-0118-0

[B32] Gong H. Zhu X. Chen K. Wang S. Zhang C. (2005). Silicon alleviates oxidative damage of wheat plants in pots under drought. Plant Sci. 169, 313–321. doi: 10.1016/j.plantsci.2005.02.023

[B33] Hamayun M. Sohn E. Y. Khan S. A. Shinwari Z. K. Khan A. L. Lee I. J. (2010). Silicon alleviates the adverse effects of salinity and drought stress on growth and endogenous plant growth hormones of soybean (*Glycine max* l.). Pak. J. Bot. 42, 1713–1722.

[B34] Hannan A. Hoque M. N. Hassan L. Robin A. H. K. (2020). “Adaptive mechanisms of root system of rice for withstanding osmotic stress,” in Recent advances in rice research. Ed. Ansari M. U. R. (London: IntechOpen). doi: 10.5772/intechopen.93815

[B35] Heath R. L. Packer L. (1968). Photoperoxidation in isolated chloroplastsasts. I. Kinetics and stoichiometry of fatty acid peroxidation. Arch. Biochem. Biophys. 125, 189–198. doi: 10.1016/0003-9861(68)90654-1 5655425

[B36] Henry A. Wehler R. Grondin A. Franke R. Quintana M. (2016). Environmental and physiological effects on grouping of drought-tolerant and susceptible rice varieties related to rice (*Oryza sativa*) root hydraulics under drought. Ann. Bot. 118, 711–724. doi: 10.1093/aob/mcw068 27192712 PMC5055623

[B37] Huang Z. A. Jiang D. A. Yang Y. Sun J. W. Jin S. H. (2004). Effects of nitrogen deficiency on gas exchange, chlorophyll fluorescence, and antioxidant enzymes in leaves of rice plants. Photosynthetica 42, 357–364. doi: 10.1023/B:PHOT.0000046153.08935.4c

[B38] Hussain S. Mumtaz M. Manzoor S. Shuxian L. Ahmed I. Skalicky M. . (2021). Foliar application of silicon improves growth of soybean by enhancing carbon metabolism under shading conditions. Plant Physiol. Biochem. 159, 43–52. doi: 10.1016/j.plaphy.2020.11.053 33338819

[B39] Jackson M. L. (1973). Soil chemical analysis (New Delhi: Prentice Hall India Pvt. Ltd.), 498 pp.

[B40] Javaid M. H. Khan A. R. Salam A. Neelam A. Azhar W. Ulhassan Z. . (2022). Exploring the adaptive responses of plants to abiotic stresses using transcriptome data. Agriculture 12, 211. doi: 10.3390/agriculture12020211

[B41] Joseph E. A. Radhakrishnan V. V. Mohanan K. V. (2015). A study on the accumulation of proline- an osmoprotectant amino acid under salt stress in some native rice cultivars of north kerala, India. Univers. J. Agric. Res. 3, 15–22. doi: 10.13189/ujar.2015.030104

[B42] Kadam N. N. Yin X. Bindraban P.S. Paul Struik B. P.C. Jagadish K.S.V. (2015). Does morphological and anatomical plasticity during the vegetative stage make wheat more tolerant of water deficit stress than Rice? Plant Physiol. 167, 1389–1401. doi: 10.1104/pp.114.253328 25614066 PMC4378155

[B43] Khedr R. A. Sorour S. G. R. Aboukhadrah S. H. El Shafey N. M. Abd Elsalam H. E. El-Sharnouby M. E. . (2022). Alleviation of salinity stress effects on agro-physiological traits of wheat by auxin, glycine betaine, and soil additives. Saudi J. Biol. Sci. 29, 534–540. doi: 10.1016/j.sjbs.2021.09.027 35002449 PMC8717150

[B44] Kikuta M. Makihara D. Arita N. Miyazaki A. Yamamoto Y. (2016). Growth and yield responses of upland NERICAs to variable water management under field conditions. Plant Prod. Sci. 20, 36–46. doi: 10.1080/1343943X.2016.1245102

[B45] Kim Y. Chung Y. S. Lee E. Tripathi P. Heo S. Kim K. H. (2020). Root response to drought stress in rice (*Oryza sativa* l.). Int. J. Mol. Sci. 21, 1513. doi: 10.3390/ijms21041513 32098434 PMC7073213

[B46] Kumar S. Dey P. (2011). Effects of different mulches and irrigation methods on root growth, nutrient uptake, water-use efficiency and yield of strawberry. Sci. Hortic. 127, 318–324. doi: 10.1016/j.scienta.2010.10.023

[B47] Lee S. S. Shah H. S. Awad Y. M. Kumar S. Ok Y. S. (2015). Synergy effects of biochar and polyacrylamide on plants growth and soil erosion control. Environ. Earth Sci. 74, 2463–2473. doi: 10.1007/s12665-015-4262-5

[B48] Lemoine R. La Camera S. Atanassova R. Dédaldéchamp F. Allario T. Pourtau N. . (2013). Source-to-sink transport of sugar and regulation by environmental factors. Front. Plant Sci. 4. doi: 10.3389/fpls.2013.00272 PMC3721551 23898339

[B49] Liang Y. Sun W. Zhu Y. G. Christie P. (2007). Mechanisms of silicon-mediated alleviation of abiotic stresses in higher plants: A review. Environ. pollut. 147, 422–428. doi: 10.1016/j.envpol.2006.06.008 16996179

[B50] Li Z. Song Z. Yan Z. Hao Q. Song A. Liu L. . (2018). Silicon enhancement of estimated plant biomass carbon accumulation under abiotic and biotic stresses. a meta-analysis. Agron. Sustain. Dev. 38, 26. doi: 10.1007/s13593-018-0496-4

[B51] Lum M. S. Hanafi M. M. Rafii Y. M. Akmar A. S. N. (2014). Effect of drought stress on growth, proline and antioxidant enzyme activities of upland rice. J. Anim. Plant Sci. 25, 1487–1493.

[B52] Malik M.A. Wani A.H. Mir S.H. Rehman I.U Tahir I. Ahmad P . (2021). Elucidating the role of silicon in drought stress tolerance in plants. Plant Physiol. Biochem. 165, 187–195. doi: 10.1016/j.plaphy.2021.04.021 34049031

[B53] Moran R. (1982). Formulae for determination of chlorophyllous pigments extracted with N,N-dimethylformamide. Plant Physiol. 69, 1376–1381. doi: 10.1104/pp.69.6.1376 16662407 PMC426422

[B54] Muthayya S. Sugimoto J. D. Montgomery S. Maberly G. F. (2014). An overview of global rice production, supply, trade, and consumption. Ann. N. Y. Acad. Sci. 1324, 7–14. doi: 10.1111/nyas.12540 25224455

[B55] Naiem S. Y. Badran A. E. Boghdady M. S. Aljuaid B. S. El-Shehawi A. M. Salem H. M. . (2022a). Performance of some elite potato cultivars under abiotic stress at north Sinai. Saudi J. Biol. Sci. 29, 2645–2655. doi: 10.1016/j.sjbs.2021.12.049 35531158 PMC9072938

[B56] Naiem S. Y. Badran A. E. Boghdady M. S. Alotaibi S. S. El-Shehawi A. M. Salem H. M. . (2022b). Stability and anatomical parameters of irradiated potato cultivars under drought stress. Saudi J. Biol. Sci. 29, 2819–2827. doi: 10.1016/j.sjbs.2022.01.003 35531191 PMC9073068

[B57] Panda D. Mishra S. S. Behera P. K. (2021). Drought tolerance in rice: Focus on recent mechanisms and approaches. Rice Sci. 28, 119–132. doi: 10.1016/j.rsci.2021.01.002

[B58] Pandey V. Shukla A. (2015). Acclimation and tolerance strategies of rice under drought stress. Rice Sci. 22, 147–161. doi: 10.1016/j.rsci.2015.04.001

[B59] Prasad V. B. R. Govindaraj M. Djanaguiraman M. Djalovic I. Shailani A. Rawat N. . (2021). Drought and high temperature stress in sorghum: Physiological, genetic, and molecular insights and breeding approaches. Int. J. Mol. Sci. 22, 9826 doi: 10.3390/ijms22189826 34575989 PMC8472353

[B60] Pütter J. (1974). “Peroxidases,” in Methods of enzymatic analysis. Ed. Bergmeyer H. U. (Weinhan: Verlag Chemie), 685–690.

[B61] Ranathunge K. Lin J. Steudle E. Schreiber L. (2011). Stagnant deoxygenated growth enhances root suberization and lignifications, but differentially affects water and NaCl permeabilities in rice (*Oryza sativa* l.) roots. Plant Cell Environ. 34, 1223–1240. doi: 10.1111/j.1365-3040.2011.02318.x 21414017

[B62] R Core Team . (2021). R: A language and environment for statistical computing (Vienna, Austria: R Foundation for Statistical Computing). Available at: https://www.R-project.org/ (Accessed August 15, 2022).

[B63] Romero-Aranda M. R. Jurado O. Cuartero J. (2006). Silicon alleviates the deleterious salt effect on tomato plant growth by improving plant water status. J. Plant Physiol. 163, 847–855. doi: 10.1016/j.jplph.2005.05.010 16777532

[B64] Ruzin S. E. (1999). Plant (New York: Oxford University Press), 322.

[B65] Salisbury F. B. Ross C. W. (1992). Plant physiology (Belmont, USA: Wadsworth Publishing Company), 682 pp.

[B66] Sikuku P. A. Onyango J. C. Netondo G. W. (2012). Physiological and biochemical responses of five nerica rice varieties (*Oryza sativa* l.) to water deficit at vegetative and reproductive stages. Agr. Biol. J. N. Am. 3, 93–104. doi: 10.5251/abjna.2012.3.3.93.104

[B67] Simova-Stoilova S. Demirevska K. Petrova T. Tsenov N. Feller U. (2008). Antioxidative protection in wheat varieties under severe recoverable drought at seedling stage. Plant Soil Environ. 54, 529–536. doi: 10.17221/427-PSE

[B68] Singh B. Reddy K. R. Redoña E. D. Walker T. (2017). Screening of rice cultivars for morpho-physiological responses to early-season soil moisture stress. Rice Sci. 24, 322–335. doi: 10.1016/j.rsci.2017.10.001

[B69] Sonobe K. Hattori T. An P. Tsuji W. Eneji A. E. Kobayashi S. . (2011). Effect of silicon application on sorghum root responses to water stress. J. Plant Nutr. 34, 71–82. doi: 10.1080/01904167.2011.531360

[B70] Soylemezoglu G. Demir K. Inal A. Gunes A. (2009). Effect of silicon on antioxidant and stomatal response of two grapevine (*Vitis vinifera* l.) rootstocks grown in boron toxic, saline and boron toxic-saline soil. Sci. Horti. 123, 240–246. doi: 10.1016/j.scienta.2009.09.005

[B71] Sullivan C. Y. Ross W. M. (1979). “Selecting for drought and heat resistance in grain sorghum,” in Stress physiology in crop plants. Eds. Mussell H. Staples R. C. (New York: John Wiley and Sons), 263–281.

[B72] Tabachnick B. G. Fidell L. S. (1996). Using multivariate statistics (New York: HarperCollins College Publishers), 880 pp.

[B73] Tian Z. Wang F. Zhang W. Liu C. Zhao X. (2012). Antioxidant mechanism and lipid peroxidation patterns in leaves and petals of marigold in response to drought stress. Hortic. Environ. Biotechnol. 53, 183–192. doi: 10.1007/s13580-012-0069-4

[B74] Usman M. Raheem Z. F. Ahsan T. Iqbal A. Sarfaraz Z. N. Haq Z. (2013). Morphological, physiological and biochemical attributes as indicators for drought tolerance in rice (*Oryza sativa* l.). Europ. J. Biol. Sci. 5, 23–28. doi: 10.5829/idosi.ejbs.2013.5.1.1104

[B75] Zewdie S. Mats O. Fetene M. (2007). Growth, gas exchange, chlorophyll a fluorescence, biomass accumulation and partitioning in droughted and irrigated plants of two enset (*Ensete ventricosum* welw. cheesman) clones. J. Agron. 6, 499–508. doi: 10.3923/ja.2007.499.508

[B76] Zhang Y. Luan Q. Jiang J. Li Y. (2021). Prediction and utilization of malondialdehyde in exotic pine under drought stress using near-infrared spectroscopy. Front. Plant Sci. 12. doi: 10.3389/fpls.2021.735275 PMC8558207 34733301

