# Supplementary material for: Foliar spray of silica improved water stress tolerance in rice (Oryza sativa L.) cultivars
Source: Front Plant Sci. 2022 Nov 16;13:935090. doi: 10.3389/fpls.2022.935090 (PMC9709440; doi:10.3389/fpls.2022.935090)
Supplement: Supplementary file 1 [file DataSheet_1.pdf]

Supplementary material

Foliar spray of silica improved water stress tolerance in rice (*Oryza sativa* L.) cultivars

Samira A.F. El-Okkiah, Mohamed M. El-Afry, Safaa S. Eldin, Amira M. El-Tahan, Omar M. Ibrahim, Mostafa M. Negm, Mohamad Alnafissa, Mohamed T. El-Saadony, Khaled A. El-Tarabily*, Synan F. AbuQamar* and Dalia A. Selim

*** Correspondence:**

Khaled A. El-Tarabily
ktarabily@uaeu.ac.ae

Synan F. Abu Qamar
sabuqamar@uaeu.ac.ae

Supplementary material

Table S1. Physical and chemical properties of the experimental site during the 2019 and 2020 seasons.

Table S2. The pedigree, salience and feature of rice genotypes used in the study.

Table S3. Effects of foliar application of silica on some morphological parameters (plant height, flag leaf area and root volume) at flowering stage in response to drought stress during the growing seasons of 2019 and 2020.

Table S4. Changes in stem xylem, endodermis cell wall and sclerenchyma layer thickness in well-watered and drought stressed rice (four months old) plants.

Table S5. Effects of foliar application of silica on transverse sections of stems the three rice cultivars in response to drought stress.

Figure S1. Transverse sections of stems of rice cultivars affected by silica application and drought stress. Giza178 and Sakha102 were treated with or without silica under drought stress conditions. Response of rice cultivar (**A**) Si0+Giza178 to well-watered irrigation (control) conditions. (**B**) Si0+Giza178; (**C**) Si0+Sakha102; and (**D**) Si200+Giza178 under drought stress conditions. Bars = 200 µm. Stems were stained with safranin and fast green. Si, silica; Si0, 0 mg l^{-1} Si; Si200, 200 mg l^{-1} Si; control, well-watered (80% water-holding capacity (WHC); drought, 40% WHC; MX, metaxylem; FP, fundamental parenchyma; CF, cortical fiber; SVB, small vascular bundle.

Table S1. Physical and chemical properties of the experimental site during the 2019 and 2020 seasons.

Chemical analyses		2019	2020
**pH**		8.05	7.99
**Electrical conductivity dSm^{-1}**		2.39	2.33
**Cations**	**K^{+}**	0.25	0.25
	**Na^{+}**	12.87	12.54
	**Ca^{++}**	8.70	8.48
	**Mg^{++}**	1.77	1.74
**Anions**	**HCO_3^{-}**	4.13	4.03
	**Cl^{-}**	13.24	12.9
	**SO_4^{--}**	6.18	6.03

Cations and anions are measured in meq 100 g $soil^{-1}$.

Table S2. The pedigree, salience and feature of rice genotypes used in the study.

Genotype	Pedigree	Salience and feature
**Sakha102**	GZ4096-7-1/GZ4120-2-5-2 (Giza 177)	Japonica type – sensitive to heat – short stature, early duration- resistance to blast.
**Sakha107**	Giza 177/BLI	Japonica type – tolerant to heat – short stature- early duration- resistant to blast.
**Giza178**	Giza175/Milyang 49	Indica/japonica type, medium maturing, semi-dwarf, resistant to blast, medium grain, tolerant to heat and high yield.

The three rice cultivars used were Giza178, Sakha102, and Sakha107.

Table S3. Effects of foliar application of silica (Si) on some morphological parameters (plant height, flag leaf area and root volume) at flowering stage in response to drought stress during the growing seasons of 2019 and 2020.

Water treatments	Silica (mg l^{-1}) treatments	Cultivars	Plant height (cm)		Flag leaf area (cm^2)		Root volume (cm^3)	
			2019	2020	2019	2020	2019	2020
Well-irrigated (80% WHC)	Si0	G178	117.00^{de}	115.67^{de}	24.23^{bcd}	24.50^{bcd}	65.00^{cd}	67.67^{de}
	Si0	SK102	105.66^{ef}	104.33^{ef}	21.87^{def}	22.24^{c-e}	42.50^{hij}	47.67^{i}
	Si0	SK 107	108.83d^{ef}	107.5d^{ef}	25.33^{bc}	23.02^{c-e}	51.67^{fgh}	52.00^{g-i}
	Si 200	G178	122.00^{b}	120.67^{b}	30.17^{a}	29.71^{a}	77.67^{ab}	82.50^{ab}
	Si 200	SK102	113.66^{fg}	112.33^{fg}	25.83^{b}	25.53^{bc}	47.67^{I}	52.00^{g-i}
	Si 200	SK 107	118.00^{cde}	116.67^{cd}	20.20^{d-g}	21.23^{def}	67.67^{bc}	69.00^{d}
	Si 400	G178	122.83^{b}	121.50^{b}	24.60^{bcd}	25.65bc	82.67^{a}	77.83^{bc}
	Si 400	SK102	112.4g^{h}	111.00^{f}	22.33^{c-e}	23.33c-e	55.00^{d-g}	62.50^{def}
	Si 400	SK 107	119.35^{de}	118.00^{d}	24.47^{bcd}	25.07bc	52.67^{efgh}	57.50^{fgh}
Drought (40% WHC)	Si0	G178	101.34^{hi}	100.00^{h}	20.63^{d}	19.69ef	57.50^{c-f}	60.00^{efg}
	Si0	SK102	95.50^{1i}	94.00^{i}	14.67^{i}	15.07hi	27.67^{k}	32.33^{jk}
	Si0	SK 107	98.40^{gh}	97.00^{hi}	18.83^{efg}	18.12efg	37.67^{ijk}	45.00^{i}
	Si 200	G178	113.66^{gh}	112.33^{fg}	22.66^{c-e}	23.33^{c-e}	78.33^{ab}	82.67^{ab}
	Si 200	SK102	103.66^{efg}	102.33^{gh}	16.30^{gh}	17.15^{e-h}	43.33^{hij}	45.00^{i}
	Si 200	SK 107	108.33^{def}	107.00^{ef}	20.47^{de}	19.07^{d-g}	63.33^{cde}	65.00^{def}
	Si 400	G178	124.33^{a}	123.00^{a}	21.87^{def}	22.24^{d-e}	65.00^{cd}	70.00^{cd}
	Si 400	SK102	116.23^{def}	114.90^{de}	17.27^{ef}	15.92^{hij}	45.00^{ghi}	50.00^{hi}
	Si 400	SK 107	122.93^{b}	121.6^{b}	20.20^{d-g}	21.23^{def}	55.00^{defg}	62.6^{def}
LSD 0.05			5.75	5.14	5.14	5.75	11.65	8.08

Within columns, values followed by the same letter are not significantly (P>0.05) different. Si, silica; Si0, 0 mg l^{-1} Si; Si200, 200 mg l^{-1} Si; Si400, 400 mg l^{-1} Si; WHC, water-holding capacity. The three rice cultivars used were Giza178, Sakha102, and Sakha107.

Table S4. Changes in stem xylem, endodermis cell wall and sclerenchyma layer thickness in well-watered and drought stressed rice (four months old) plants (no silica application).

Cultivar	Xylem cell wall		Endoderm (μm)		Sclerenchyma layer	
	Well-	Drought	Well-	Drought	Well-	Drought
**Giza178+Si0**	4.6[a]	8.9[a]	5.4[a]	10.4[a]	1.5[a]	3.8[ab]
**Sakha102+Si0**	3.7[c]	5.5[c]	4.7[b]	7.2[c]	1.2[a]	2.8[c]
**Sakha107+Si0**	4.4[ab]	6.8[b]	5.2[ab]	9.3[b]	1.5[a]	4.2[a]
**Giza178+Si400**	4.5[a]	9.0[a]	5.4[ab]	10.3[a]	1.9[a]	4.2[a]

Different letters in the same column showed significant differences ($P<0.05$). WHC, water-holding capacity; well-watered, 80% WHC (control); drought, 40% WHC. Si, silica; Si0, 0 mg l^{-1} Si; Si400, 400 mg l^{-1} Si. The three rice cultivars used were Giza178, Sakha102, and Sakha107.

Table S5. Effects of foliar application of silica on transverse sections of stems the three rice cultivars in response to drought stress.

Treatment	ET thickness	XV diameter	PhT thickness	Inner VB dimension		Fundamental parenchyma
				Thickness	Width	
**Si0+Giza178 (control)**	5.83^{b}	16.67^{c}	14.67^{b}	65.83^{b}	88.33^{c}	306.67^{b}
**Si0+Giza178**	6.67^{ab}	17.38^{b}	16.58^{a}	75.83^{a}	103.83^{a}	356.67^{a}
**Si400+Giza178**	7.50^{a}	18.33^{a}	14.17^{c}	65.00^{b}	93.33^{b}	316.83^{b}
**Si0+Sakha102**	4.82^{c}	16.33^{c}	14.31^{bc}	65.32^{b}	86.66^{d}	304.22^{b}
**Si0+Sakha102**	4.17^{d}	14.58^{d}	11.25^{d}	58.33^{c}	60.83^{e}	266.67^{c}
**Si400+Sakha102**	5.50^{b}	15.33^{d}	14.67^{b}	65.00^{b}	93.41^{b}	306.83^{b}

Different letters in the same column showed significant differences (P<0.05). Si, silicon; Si0, 0 mg l^{-1} Si; Si400, 400 mg l^{-1} Si; WHC, water-holding capacity; well-watered, 80% WHC (control); drought, 40% WHC; ET, epidermis; XV, xylem vessel; VB, vascular bundle. The three rice cultivars used were Giza178, Sakha102, and Sakha107.

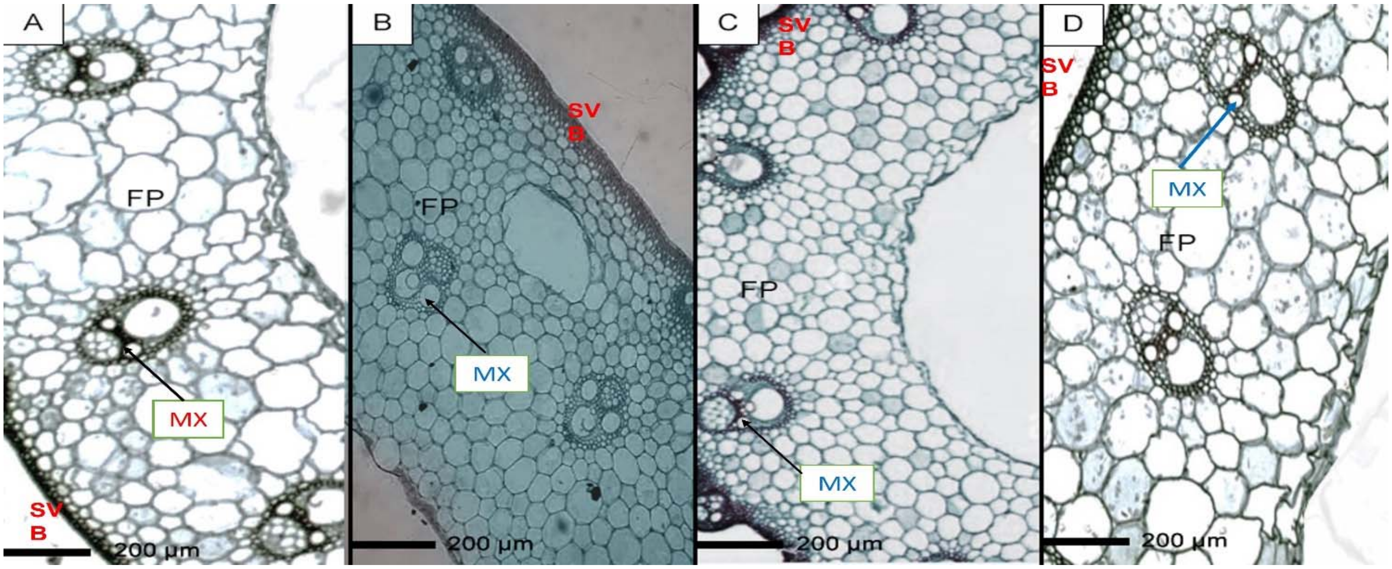

Figure S1. Transverse sections of stems of rice cultivars affected by silica application and drought stress. Giza178 and Sakha102 were treated with or without silica under drought stress conditions. Response of rice cultivar (**A**) Si0+Giza178 to well-watered irrigation (control) conditions. (**B**) Si0+Giza178; (**C**) Si0+Sakha102; and (**D**) Si200+Giza178 under drought stress conditions. Bars = 200 µm. Stems were stained with safranin and fast green. Si, silica; Si0, 0 mg l^{-1} Si; Si200, 200 mg l^{-1} Si; control, well-watered (80% water-holding capacity (WHC); drought, 40% WHC; MX, metaxylem; FP, fundamental parenchyma; CF, cortical fiber; SVB, small vascular bundle; Si, silica; Si0, 0 mg l^{-1} Si; Si200, 200 mg l^{-1} Si.
